# Functional Anatomy of Polycomb and Trithorax Chromatin Landscapes in *Drosophila* Embryos

**DOI:** 10.1371/journal.pbio.1000013

**Published:** 2009-01-13

**Authors:** Bernd Schuettengruber, Mythily Ganapathi, Benjamin Leblanc, Manuela Portoso, Rami Jaschek, Bas Tolhuis, Maarten van Lohuizen, Amos Tanay, Giacomo Cavalli

**Affiliations:** 1 Institut de Génétique Humaine, CNRS, Montpellier, France; 2 Department of Computer Science and Applied Mathematics, The Weizmann Institute of Science, Rehovot, Israel; 3 Division of Molecular Genetics, and the Centre for Biomedical Genetics, The Netherlands Cancer Institute, Amsterdam, The Netherlands; Harvard University, United States of America

## Abstract

Polycomb group (PcG) and trithorax group (trxG) proteins are conserved chromatin factors that regulate key developmental genes throughout development. In *Drosophila*, PcG and trxG factors bind to regulatory DNA elements called PcG and trxG response elements (PREs and TREs). Several DNA binding proteins have been suggested to recruit PcG proteins to PREs, but the DNA sequences necessary and sufficient to define PREs are largely unknown. Here, we used chromatin immunoprecipitation (ChIP) on chip assays to map the chromosomal distribution of *Drosophila* PcG proteins, the N- and C-terminal fragments of the Trithorax (TRX) protein and four candidate DNA-binding factors for PcG recruitment. In addition, we mapped histone modifications associated with PcG-dependent silencing and TRX-mediated activation. PcG proteins colocalize in large regions that may be defined as polycomb domains and colocalize with recruiters to form several hundreds of putative PREs. Strikingly, the majority of PcG recruiter binding sites are associated with H3K4me3 and not with PcG binding, suggesting that recruiter proteins have a dual function in activation as well as silencing. One major discriminant between activation and silencing is the strong binding of Pleiohomeotic (PHO) to silenced regions, whereas its homolog Pleiohomeotic-like (PHOL) binds preferentially to active promoters. In addition, the C-terminal fragment of TRX (TRX-C) showed high affinity to PcG binding sites, whereas the N-terminal fragment (TRX-N) bound mainly to active promoter regions trimethylated on H3K4. Our results indicate that DNA binding proteins serve as platforms to assist PcG and trxG binding. Furthermore, several DNA sequence features discriminate between PcG- and TRX-N–bound regions, indicating that underlying DNA sequence contains critical information to drive PREs and TREs towards silencing or activation.

## Author Summary

Although all cells of a developing organism have the same DNA, they express different genes and transmit these gene expression patterns to daughter cells through multiple rounds of cell division. This cellular memory for gene expression states is maintained by two groups of proteins: Polycomb-group proteins (PcG), which establish and maintain stable gene silencing, and trithorax group proteins (trxG), which counteract silencing and enable gene activation. It is unknown how this balance works and how exactly these proteins are recruited to their target sequences. By mapping the genome-wide distribution of PcG and trxG factors and proteins known to recruit them to chromatin, we found that putative PcG recruiters are not only colocalized at PcG binding sites, but also bind to many other genomic regions that are actually the binding sites of the Trithorax complex. We identified new DNA sequences important for the recruitment of both PcG and trxG proteins and showed that the differential binding of the recruiters PHO and PHOL may discriminate between active and inactive regions. Finally, we found that the two fragments of the Trithorax protein have different chromosomal distributions, suggesting that they may have distinct nuclear functions.

## Abstract 3

Comparison of the genome-wide distribution of PcG, trxG, and sequence-specific DNA binding proteins allowed the identification of key signals leading to Polycomb or Trithorax recruitment.

## Introduction

Polycomb group (PcG) and trithorax group (trxG) proteins are conserved chromatin factors that maintain, respectively, the memory of inactive or active states of homeotic genes throughout development. They also regulate many other target genes (reviewed in [1]) and misregulation of PcG and trxG genes leads to loss of cell fates, aberrant cell proliferation and tumorigenesis. Moreover, PcG and trxG factors play an important role in diverse epigenetic processes such as stem cell pluripotency and plasticity, genomic imprinting, and X chromosome inactivation [2]. In *Drosophila*, PcG and trxG proteins are recruited to chromatin by regulatory DNA elements called PcG and trxG response elements (PREs and TREs, respectively). These elements were shown to drive epigenetic inheritance of silent and active chromatin states throughout development [3,4]. Biochemical studies on PcG proteins revealed that they exist in at least three distinct multiprotein complexes (reviewed in [5]). PRC2-type complexes contain the four core components E(z) (Enhancer of zeste), Esc (Extra sex combs), Su(z)12 (Suppressor of zeste 12), and Nurf-55. The SET domain-containing E(z) subunit trimethylates lysine 27 of histone H3 (H3K27me3). This mark is specifically recognized by the chromo domain of Polycomb (PC), a subunit of the PRC1-type complex [6]. PRC1 contains PC, Polyhomeotic (PH), PSC (Posterior sex combs), and the histone H2A ubiquityltransferase dRing, in addition to several other components, including TBP-associated factors [7]. The PhoRC complexes include the sequence-specific DNA binding proteins Pleiohomeotic (PHO) or its homolog Pleiohomeotic-like (PHOL), as well as the dSfmbt protein (*Scm*-related gene containing four MBT domains). Several trxG complexes have been identified: TAC1 (Trithorax Acetylation Complex) with the histone methyltransferse Trithorax (TRX), NURF, SWI/SNF, ASH1, and ASH2 (for reviews, see [3,8]). Interestingly, the human TRX homolog MLL1 has been previously shown to be cleaved at two conserved sites by the Taspase1 enzyme, generating an N-terminal and a C-terminal fragment, which can heterodimerize [9,10]. However, it is unknown whether the two moieties can have different functions or chromosomal distributions. Additional PcG/trxG proteins have been identified that are not part of the core of these complexes, but are associated with them and, therefore, can be considered as PcG/trxG-associated proteins [11]. These proteins may exist as individual molecules in the cell, but it is also possible that they are part of other protein complexes that contain additional, as yet unidentified PcG/trxG proteins.

PcG and trxG complexes (except PhoRC) do not bind their target DNA in a sequence-specific manner in vitro, but are recruited to PRE/TRE sequences in vivo. A simple pathway for PcG protein recruitment based on stepwise recruitment of PRC2 proteins by PhoRC, followed by PRC1 recruitment by the H3K27me3 mark deposited by PRC2 has been suggested [12]. However, PcG recruitment seems to be more complex. PHO interacts with PRC2 as well as with the PC and PH subunits of PRC1 in vitro [13]. PHO/PHOL binding sites alone are insufficient to tether PcG proteins to DNA in vivo [14,15], and most PcG sites are stained normally in polytene chromosomes in *pho/phol* double mutants despite lack of detectable PHO and PHOL proteins [15]. However, PcG protein binding is lost at the bxd PRE in *pho/phol* double-mutant wing discs [12], suggesting that the role of PHO and possibly PHOL is important. Other factors have been shown to be involved in recruitment, such as GAGA factor (GAF), Pipsqueak (PSQ), Dorsal switch protein (DSP1), Zeste, Grainyhead (GH), and Sp1/KLF (reviewed in [5]). Mutations in the corresponding genes do not have a clear PcG phenotype, and intriguingly, all seem to be involved in activation as well as in repression. In summary, many unresolved questions regarding PcG recruitment still remain, and the current model proposes that a combination of several DNA binding factors, and maybe yet-unknown components, could lead to tethering of PcG proteins to DNA.

Recently, the distribution of several core components of PcG members and their associated histone modifications has been analyzed in fly as well as mammalian cells [16–22]. Yet, a comprehensive genome-wide binding map of PcG/trxG recruitment factors and of trxG proteins is still lacking. Here, we have generated high-resolution genome-wide binding maps in *Drosophila* embryos of two PRC1 components and their associated histone mark H3K27me3, the N- and the C-terminal part of the TRX protein and their associated histone mark H3K4me3 as well as four sequence-specific DNA binding proteins known to be involved in recruitment of Polycomb proteins. Our results show the complementarity between PcG and trxG protein binding in the genome and suggest that multiple DNA binding proteins participate in setting up this PcG and trxG protein distribution.

## Results

### Overview of PcG and trxG Genomic Landscapes

**Figure 1:**
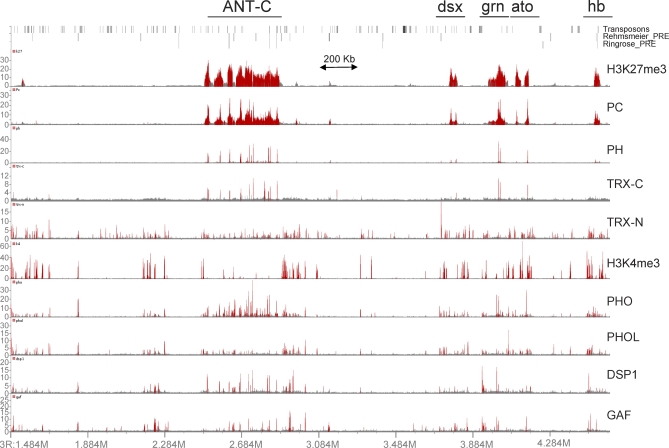
Genomic Distribution of PcG and TrxG Proteins and associated Histone Modifications in a Segment of Chromosome 3R The plots show the ratios (fold change) of specific IP versus mock IP assays along part of the chromosome 3R. Significantly enriched fragments (*p*-value < 1E−04) are shown in red. All the profiles generated are available for viewing in an interactive browser at http://purl.oclc.org/NET/polycomb. Position of genes (FlyBase annotation 4.3) is shown at the top of the figure. Transposons and previously predicted PREs (M. Rehmsmeier, personal communication; [24,25]) are indicated by gray bars. Note that PC and H3K27me3 are bound to large genomic regions, whereas the other profiles show sharp localized binding. PcG recruitment factors were bound at PREs as well as at many other promoter regions where no PcG binding is detected. The N-terminal fragment of TRX (TRX-N) shows only weak binding to PREs, but colocalizes with H3K4me3 and sequence-specific DNA binding proteins at many promoter regions. The C-terminal fragment of TRX (TRX-C) is only strongly bound at PcG binding sites. ANT-C, Antennapedia complex; *ato*, atonal; *dsx*, doublesex; *grn*, grain; *hb*, hunchback.

**Figure 2:**
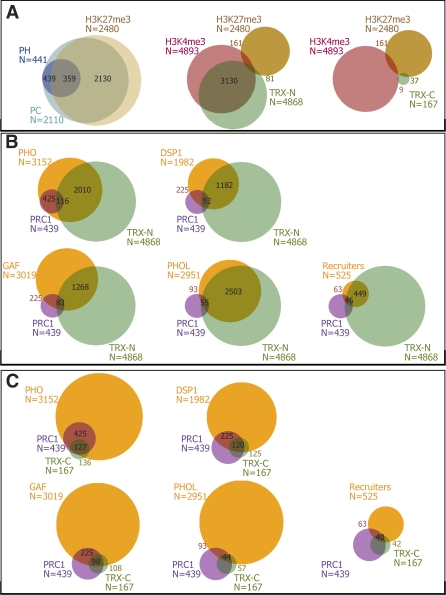
Venn Diagrams Showing Overlap between Bound Regions of Different Protein Profiles All the bound regions taken for analysis were with *p*-value < 1E−04. For PC and H3K27me3, the unstitched regions (see Text S1) were analysed. PRC1 denotes the regions cobound by PC and PH. Recruiters are the regions cobound by PHO, DSP1, GAF, and PHOL. (A) PcG binding is highly correlated. Nearly all PH sites are bound by PC and H3K27me3. Minimal overlap is seen between H3K27me3 and TRX-N/H3K4me3 or TRX-C/H3K4me3. (B) Occurrence of PcG recruitment factors along with PRC1 and TRX-N. Note that a large proportion of each factor is bound with TRX-N. Interestingly, PHO co-occurs with nearly all the PRC1 (PC+PH). PHOL minimally colocalizes with PRC1 but colocalizes extensively with TRX-N. (C) Occurrence of PcG recruitment factors along with PRC1 and TRX-C. Note the high overlap of TRX-C with PRC1.

Using chromatin immunoprecipitation (ChIP) in 4–12-h-old Drosophila melanogaster embryos coupled with genome-wide high-density tiling arrays, we mapped the distribution of the PRC1 components: PC and PH, the N- and the C-terminal part of the Trithorax protein (TRX-N and TRX-C, respectively), and the histone H3K27me3 and H3K4me3 marks. We also determined the genome-wide binding profile of GAF, PHO, PHOL and DSP1, four DNA binding proteins thought to be involved in PcG recruitment. Reproducibility of biological replicates is shown in Figures S1 and S2. Figure 1 shows an example of the different profiles along part of chromosome 3R including the HOX gene cluster named ANT-C. The statistics on the number and size of regions significantly enriched for various proteins is shown in Figures S3 and S4, and in Table S1. As observed previously, PC and H3K27me3 mark covered over 200 large domains (>5 kb), most of which contain discontinuous subregions with significant *p*-values for enrichment separated by small intervening subregions that were enriched although their *p*-values were not significant (see Text S1 for a precise definition of H3K27me3 and PC domains). The number of significantly enriched subregions for PC and H3K27me3 were 2,110 and 2,480, respectively. Nearly all PH binding sites fall into PC- and H3K27me3-bound regions (Figure 2A). The sequence-specific DNA binding proteins PHO, PHOL, DSP1, and GAF are bound to thousands of genomic sites (Table S1). Surprisingly, whereas PcG binding sites strongly predict the presence of one or more of the DNA binding factors, the converse is not true. In fact, the sequence-specific DNA binding proteins are more frequently bound to sites bound by TRX-N and trimethylated on H3K4 (see Figure 2B). Binding of the N-terminal fragment and the C-terminal fragment of TRX (TRX-N and TRX-C, respectively) correlates well at the genome-wide level (Figure S5), but the relative intensities are very different. TRX-N is significantly bound to 4,868 genomic sites, with strong binding correlated to H3K4me3-bound regions (Figure 2; a total of 4,893 regions contained H3K4me3). At most of these sites, TRX-C binding levels are higher than background, but not picked up as significant. Strong binding of TRX-C is only identified at 167 genomic sites, mainly located in PRC1-bound regions (Figure 2C) where TRX-N binds weakly if at all. All the profiles are available at an online browser at the address http://purl.oclc.org/NET/polycomb. This browser also contains data from earlier mapping studies [20,22] and from transcription profiling of staged embryos [23]. In addition, it contains the annotation of predicted PREs (M. Rehmsmeier, personal communication [24,25]), whose genomic location can be visualized along with the significantly enriched regions and with the results from our sequence analysis.

### Bivalent Domains Are Not a Common Feature of the Fly Embryo Epigenome

Recent analysis of H3K4me3 and H3K27me3 in mouse and human cells revealed the coexistence of these two marks in a large fraction of the H3K27me3 regions [26–29]. These regions encompass most of the H3K27 trimethylated sites in embryonic stem (ES) cells and a substantial portion of them in differentiated cells. Although we do frequently observe H3K4me3 occupancy at transcription start sites (TSSs) flanking PH sites, this is almost exclusively observed at the boundary of large H3K27me3 domains (see Text S1). From a total of 4,893 H3K4me3 and 2,480 H3K27me3 regions, only 161 had an overlap, i.e., only 6.5% of the H3K27me3 regions. Considering that most of the genes identified by these regions of overlap are expressed only in a fraction of the embryonic cells, we believe that most of these cases reflect a mixture of cell populations rather than true bivalency. Moreover, the H3K4me3 profile always showed sharp peaks at promoters within large H3K27me3 regions, in contrast to mammalian cells in which bivalent domains often show similar profiles with H3K4me3 and H3K27me3 spread over regions of several kilobases in size. Thus, our data suggest that H3K4me3 and H3K27me3 are generally exclusive in the fly genome. Nevertheless, individual cases of true bivalency may exist in fly embryos or at other developmental stages. A rigorous demonstration of this point will require sequential ChIP with mononucleosomal chromatin and antibodies directed against the H3K4me3 and H3K27me3 marks.

### Two Layers of Genomic Organization

**Figure 3:**
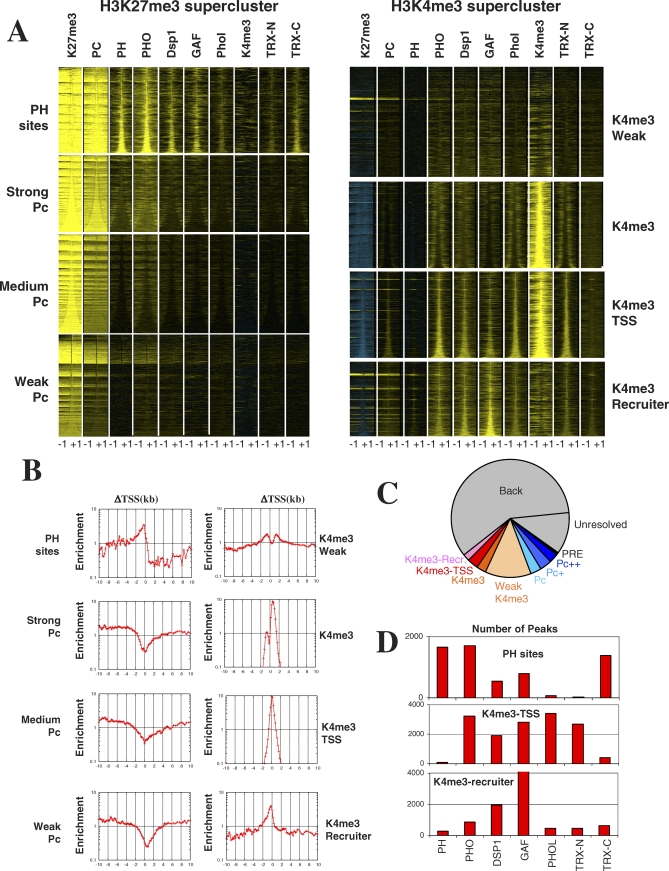
Genome-Wide Architecture of Polycomb and Trithorax Marks and Recruiters (A) Spatial clusters. We dissected our multifactor genome-wide dataset into groups of loci with common factor and histone mark occupancy (spatial clusters). Clusters are probabilistically tied together to reflect a typical genomic organization (Figure S18). Our algorithm detected two superclusters, one representing H3K27me3-marked domains (left) and the other representing H3K4me3-marked domains (right), and further decomposed each supercluster into distinct genomic behaviors. Here, we depict each cluster as a block, where rows represent the 2 kb (−1 kb to +1 kb) around cluster centers, color-coded to reflect the binding intensity of nine marks and factors (yellow indicates strong binding, blue negative enrichment). (B) We also plotted the enrichment of clusters' locations relative to the TSS (*x*-axis, zero reflect the TSS itself), normalized by the genome-wide frequency of distances from the TSS. (C) Frequency of clusters in the genome. The relative abundance of the eight clusters is shown. About two-thirds of the genome is not associated with either of our two superclusters (i.e., lboth H3K4me3 and H3K27me3 are lacking). (D) Transcription factor (TF) peaks in three clusters. We show the number of peaks (over 1.5 chip enrichment) for the PH sites, K4me3-recruiter, and K4me3-TSS clusters. The vast majority of TF peaks is observed in these three clusters, with some exceptions for GAF and TRX (unpublished data).

We sought a comprehensive characterization of the joint distribution of PcG and trxG factors and associated marks. Many of the data tracks are highly correlated among themselves (Figures S5 and S6), and are also tightly associated with other spatial genomic features like TSSs. We therefore developed a new method for dissecting a multivariate genomic profile into a hierarchy of “spatial clusters.” Briefly, “spatial clustering” can be viewed as the genomic analog of gene clustering, since it dissects the genome into clusters that share a common profile across all experimental tracks (detailed information is given in the Text S1). Unlike gene clustering, our model takes into account the genomic layout of the data, and organizes clusters spatially to probabilistically describe the typical genomic order among them. We used the clustering results (Figure 3) as a blueprint for our dataset, validating conclusions by running an independent, supervised data analysis. An example of cluster organization is illustrated in Figure S7. Analysis of the distribution of cluster location with respect to the TSS further demonstrates how the clusters are organized around genes (Figure 3B, note that TSS data were not used by the algorithm to define clusters).

As shown in Figure 3, our data reflect two levels of genomic organization. First, the genome is partitioned into three superclusters. Consistent with the mutually exclusive distribution of H3K27me3 and H3K4me3, unsupervised spatial clustering identifies a “H3K27me3-marked” supercluster and “H3K4me3-marked” supercluster, in addition to regions with no particular epigenomic enrichment (“background” supercluster, not shown in Figure 3). Second, each supercluster is subdivided into distinct clusters, and the model identifies the connections between clusters that organize the entire genome (Figure S18). The H3K27me3 superclusters are anchored around clusters characterized by high levels of PH binding (labeled as “PH sites”). These clusters include also strong PHO enrichment, presence of the recruiter factors GAF and DSP1 and TRX-C occupancy. All of the PH site clusters in the BX-C, the ANT-C, the *ph*, the *hh*, and the *en* genes were previously identified as PREs, suggesting that in general, most of the PH clusters are indeed PREs. The H3K27me3 supercluster also included three clusters with lower levels of PC and a general lack of PH and cofactors. We labeled them as “Strong,” “Medium,” and “Weak” PC clusters.

Similarly, the H3K4me3-marked supercluster was subdivided by the algorithm into four clusters. These clusters reflect clear organization around annotated TSSs, as identified by their TSS enrichment statistics (Figure 3B) and binding preferences (Figure 3D). We denoted the cluster with the most 5′ enrichment as the “K4me3-recruiters” cluster. It is characterized by high levels of GAF, DSP1, and significant, but weaker levels of PHO and PHOL, as well as medium to weak H3K4me3 levels. Enriched exactly at the TSS is the “K4me3-TSS” cluster with high H3K4me3 levels in combination with high levels of TRX-N, PHO and PHOL. The K4me3 cluster has only high levels of H3K4me3 and represent the region downstream the TSS, whereas the “weak K4me3” cluster shows low, but significant levels of H3K4me3 alone and is more weakly enriched around TSSs.

### Polycomb Domain Plasticity

PC and H3K27me3 were bound in large regions, often greater than 5 kb, with the largest ones spanning several hundred kilobases (see Figures 1 and S4A). Globally, H3K27me3 and PC profiles were very well correlated, facilitating the definition of PC domains (see Text S1), underscoring the significance of the H3K27me3 supercluster (Figure 3) identified by spatial clustering. A similar pattern was observed for PC and H3K27me3 by Schwartz et al. [20] in their genome-wide mapping studies in S2 cells and by Tolhuis et al. who used Kc cells [22].

**Figure 4:**
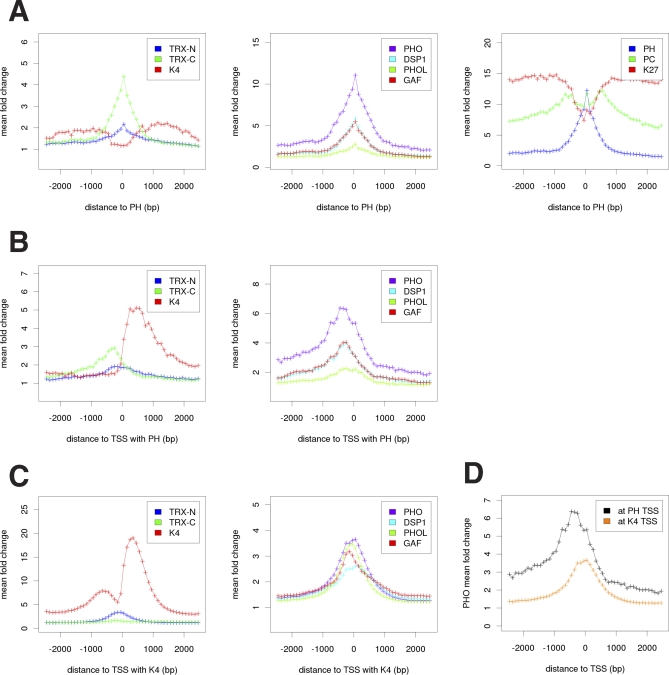
Average Chromatin Profiles at PH Sites and Transcription Start Sites (A) Shown are average fold changes of selected factors around PH local maxima (100-bp intervals in a 2.5-kb flanking region). Note the dip in values of H3K27me3 and PC at PH peaks and the stronger binding of PHO and TRX-C compared to other recruiters and TRX-N, respectively. (B and C) We classified annotated TSS (FlyBase 4.3) according to the existence of a nearby PH site (B) or H3K4me3 local maximum (C). Shown are the average fold changes for selected factors around such TSSs (in intervals of 100 bp [for PH] and 50 bp [for H3K4me3]). Note the strong binding of PHO and TRX-C and the lack of PHOL binding at PH-associated TSS. The shoulder of the H3K4me3 peak in Figure 4C (left panel) likely corresponds to promoter regions of divergently transcribed genes, because we generally do not detect H3K4me3 enrichment 5′ of the TSS of isolated genes. (D) Average fold change of PHO at TSS associated either with PH or H3K4me3.

Nearly all PH peaks were specific to PC and H3K27me3 regions (the PH sites; Figure 3) and were present in all the earlier characterized PREs. The average distribution of H3K27me3 around PH peaks takes a dip at the PH sites (Figure 4A), which may be due to nucleosome depletion at the PREs [20]. The distribution of the domain size, number of PH peaks, and genes in H3K27me3 domains is shown in Figure S4 (for an identification of candidate PcG target genes, see Text S1 and Table S2).

Despite these common features, there are differences in the positions of many of the PcG domains in different biological samples. Although the majority of our 217 H3K27me3 domains also exists in S2 cells, 79 (36%) of them did not overlap any bound regions in S2 cells. These data are corroborated by the analysis of the distribution of the PC protein which, similar to H3K27me3, forms large domains. In general, H3K27me3 differences between embryos and S2 cells paralleled differences in PC binding. The same was observed in a comparison between ChIP on chip binding of PC from embryos and the PC DamID profile obtained previously in Kc cells [22]. Interestingly, a substantial portion of the PC domains in Kc cells differed from those observed both in embryos and in S2 cells. Thus, many common PC domains are identified in various cell types, but a significant subset of them is cell-type specific rather than constitutive. These data are in agreement with previous studies suggesting that part of the PcG binding is cell-type and developmental-stage specific [19,30].

### PH Sites and the Distribution of Putative PcG Recruitment Factors

To gain more insight into PRC1 recruitment to chromatin, we examined the distribution of PcG recruitment factors at PH sites that are also bound by PC (PRC1 sites). The combination of different PcG recruitment factors at the PRC1 sites as compared to the genome is listed in Table S3 and shown in Figure 2B. Most PH binding peaks colocalize with the PcG recruitment factor PHO (96.4%) (see Figure 2B and Table 1). DSP1 and GAF were present in about 50% of the PH sites. In contrast, PHOL binding was not common at PH sites, with a frequency (21.1%) comparable to that of TRX-N (26.5%). Surprisingly, only a minority of all recruitment factors binding sites (3.2% to 13.5%) was restricted to PH sites (Table 1). Comparison with previously published Zeste data [31] showed that a moderate 25% of the Zeste sites colocalized with PH peaks. Together these data suggest a correlation gradient between different recruiters and PREs, with PHO > DSP1/GAF > Zeste/PHOL.

**Table 1:**
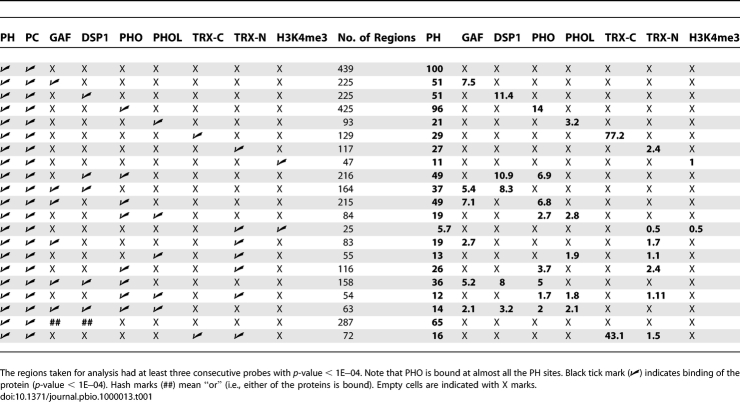
Various Combinations of Recruiter Proteins Present at PH- and PC-Bound Regions

### H3K4me3 and the Distribution of Putative PcG Recruitment Factors

The K4me3-recruiter cluster (including strong GAF and DSP1 and medium to weak H3K4me3 levels) is located in a position just upstream to the TSS. The K4me3-TSS cluster (high H3K4me3 levels and strong TRX-N, PHO, and PHOL binding) is usually following it and is almost exclusively observed over the 2 kb around the TSS. Finally, the K4me3 cluster (high H3K4me3 levels without TF occupancy) is enriched 3′ to the TSS. This organization suggests that binding of GAF and DSP1 can promote the activation of a TSS upon binding of TRX-N and the PHO/PHOL factors. Therefore, PH target promoters are strongly bound by PHO and TRX-C and depleted of PHOL and TRX-N (Figure 4B), whereas H3K4me3 promoters are bound by PHO, PHOL, and TRX-N (Figure 4C). Notably, the positions of PHO (and PHOL) in the second class of promoters is right at the TSS, whereas at PH-bound promoters, PHO is colocalized with PH upstream to the TSS (Figure 4D). This different architecture may contribute to PH recruitment or to silencing of PH-bound promoters.

### TRX Binding and Associated Histone Marks

We further analyzed active promoters and PREs/TREs by analyzing TRX binding. The human TRX homolog MLL1 is cleaved by Taspase1, generating an N-terminal and a C-terminal fragment, which can heterodimerize in vitro [9,10]. Low levels of TRX-N co-occupied PH binding sites in about 26.5% of cases (Figure 4A; Table1). However, TRX-N is present at thousands of other genomic sites, where no PcG binding can be observed. These genomic sites correspond mainly to annotated 5′ ends of genes carrying H3K4me3 peaks slightly offset towards the body of the gene in comparison to TRX-N (cluster K4me3-TSS; see also Figure 4C). Interestingly, although the TRX-C profile overall looks similar to the TRX-N, its relative binding intensities are different. TRX-C is strongly bound at PcG binding sites, whereas low binding is observed at most promoter regions of non-PcG target genes (Figure 4). These results suggest that whereas the distribution of the N-terminal part of TRX follows a general transcription cofactor role, the C-terminal part is specifically linked to PcG function. PcG proteins might repress transcription by anchoring the C-terminal portion of TRX at PREs. On the other hand, constitutive TRX-C binding at PREs/TREs might allow PcG target genes to switch their state upon strong transcriptional induction.

### Sequence Motifs Defining PH Sites and H3K4me3-Marked Clusters

**Figure 5:**
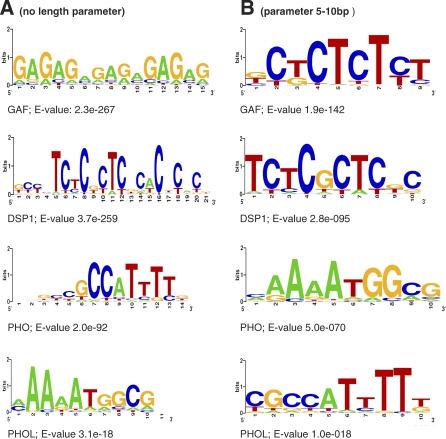
Overrepresented Sequence Motifs of PcG Recruitment Factors in ChIP on Chip Bound Regions Genome Wide (A) Overrepresented DNA motifs of GAF, DSP1, PHO, and PHOL (No motif-length parameter) (B) Overrepresented DNA motifs of DSP1, DSP1, PHO, and PHOL (motif-length parameter 5–10 bp). Sequence logo representation of the consensus is shown for top motif of each profile. MEME E-value for the motif is given below the name of the factor. Note that even though PHO and PHOL regions have the same overrepresented motif, the motif in PHOL is weakly enriched and may be a consequence of the basal PHOL-PHO overlap.

In the case of PHO, PHOL, and GAF, sequence-specific DNA binding in vitro has been shown previously [32,33]. By analyzing the collection of statistically significant bound sites for each of these proteins with the Multiple EM for motif Elicitation (MEME) algorithm, we detected the expected binding sites (Figure 5A and 5B, and Tables S6 and S7), whereas for Dsp1 [14,34], the results were not conclusive. The “GAAAA” motif was not strongly enriched among the genomic binding sites for this protein, although a degenerated GAAAA motif was found at DSP1-bound as well as at PHO- and PH-bound regions (Figures S8–S11, see Text S1 for a detailed discussion).

**Figure 6:**
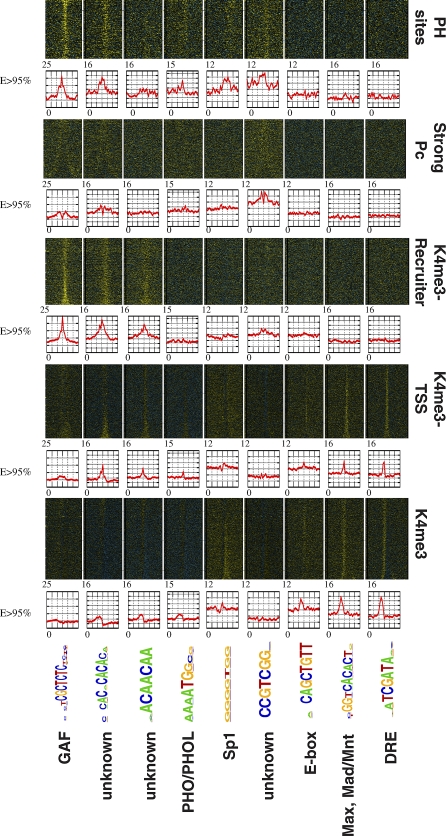
Overrepresented Sequence Motifs in the Different Spatial Clusters Shown are data for motifs that distinguish clusters or groups of clusters. The motifs were identified with no prior assumptions, but include the known GAF site [32]; PHO site [33]; Sp1/KLF site [38]; E-box [37] Max, Mad/Mnt site; and DRE site [36]. For each inferred position weight matrix (PWM), we computed the predicted binding energy for bins of 100 bp [35] and plotted a color-coded representation of it in the 8 kb around the center of each cluster (yellow indicates stronger binding). We polarized the clusters according to the strand of the nearest TSS. For each motif and cluster, we also plotted the percentage of probes with predicted binding strength in the top 5% (*y*-axis) in the 6 kb around the clusters' centers (*x*-axis).

In order to determine whether other sequence features may characterize PREs specifically, we further developed the unsupervised spatial clustering methodology (Figure 3) to allow discovery of sequence motifs that discriminate among clusters or groups of clusters. As shown in Figure 6, we discovered several known and novel motifs that are either shared among clusters or distinguish them. We visualize these results in terms of the affinities (or predicted binding energies) of the inferred position weight matrices (PWMs) in and around each our spatial clusters [35].

Two motifs (GAGA and the CA repeat motif) are marking clearly the PH sites and the K4me3-recruiter clusters. Three additional motifs are strongly marking the K4me3-TSS cluster and clearly discriminating it from the spatially coupled K4me3-recruiter cluster sites. Two of them are motifs bound by the Myc, Max, and Mad/Mnt proteins [36] and include the DNA replication element (DRE) TATCGATA, which is also consensus for several other factors including the TRF2n, Cut, and Beaf-32. The third motif (CAGCTG) is an E-box bound by bHLH proteins [37] which, like DREs, are involved in the regulation of many developmental genes. We note that the detected motif enrichments are specific to the K4me3-TSS cluster and not to general TSSs in the genome since general non–H3K4me3-associated TSSs lack these motifs.

Importantly, we also discovered motifs that discriminate between K4me3-recruiters and PH sites. The CAACAACAA motif is enriched around K4me3-recruiters, but not in and around PH sites (see also Figure S8). On the other hand, the CCGTCGG and the Sp1/KLF-like [38] GGGGTGGG motifs are specific to PH sites and not K4me3-recruiters (see also Figure S11). These motifs constitute candidates to recruit new DNA-binding factors to PREs.

In addition to these motifs, the consensus sites for PHO/PHOL, DSP1, and GAF are more strongly enriched at the 300-bp core regions around the maximal binding peak of PH than around the other genomic regions bound by the factors without PH (Table S5–S8). Thus, the density of binding sites is specific to PREs, suggesting that cooperative binding may help recruit PcG proteins. Consistent with this idea, the fold enrichment for each of the factors (with the exception of PHOL, see below) is higher at PH-bound regions compared to non–PH-bound regions (Figures S12 and 4).

Of particular interest is the distribution of the PHO motif around PH sites and the K4me3-TSS clusters. Unlike the GAGA (or CACA) motif, the frequency of motifs with sequence similarity to consensus PHO motifs is high, but these motifs are not well localized at PH sites. High predicted PHO affinities (defined by PWMs; see Text S1) were also present in the strong PcG clusters surrounding PH sites. This pattern matches perfectly with our ChIP data, which also suggest that PHO levels are regionally high around PH sites. In contrast to this pattern, the K4me3-TSS cluster is characterized by weak, but significant peaks of PHO motifs that were localized right at the TSS. This pattern is again matched by the PHO and PHOL ChIP data at the TSS of H3K4me3 associated promoters (Figure 4C).

### Discrimination between PH Sites and H3K4me3-Marked Clusters by Differential PHO and PHOL Binding

**Figure 7:**
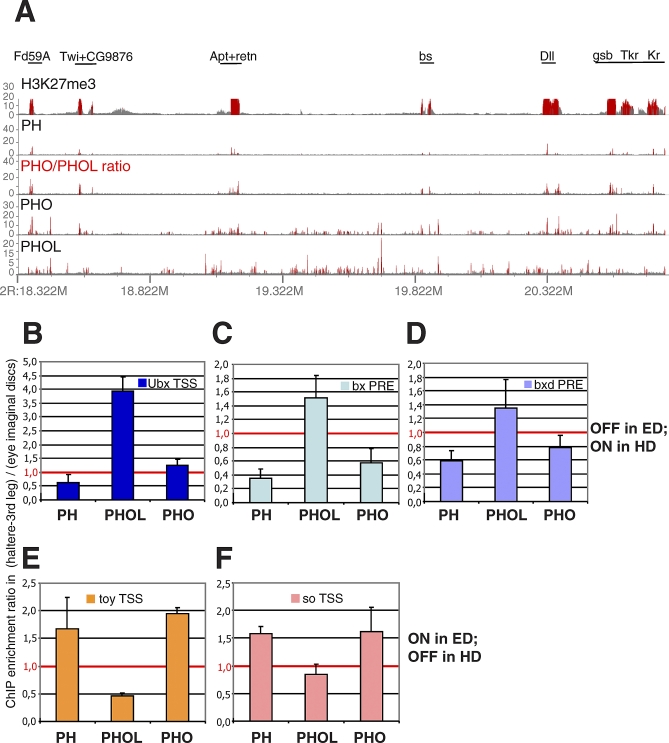
Differential PHO and PHOL Binding Ratios at PcG Target Genes in ON and OFF States (A) Profiles of H3K27me3, PH, the PHO/PHOL ratio, PHO, and PHOL are shown along part of chromosome 2R. Significantly enriched fragments (*p*-value <1E−04) are shown in red. Note that at PcG binding sites, the PHO/PHOL ratio is significantly increased. *Apt*, *apontic*; *bs*, *blistered*; *Dll*, *Distal-less*; *fd59A*, *forkhead domain 59A*; *gsb*, *gooseberry*; *Kr*, *Kruppel*; *retn*, *retained*; *Tkr*, *Tyrosine kinase-related protein*; *Twi*, *twist*. (B–F) ChIP-qPCR performed with PH, PHO, and PHOL antibodies of haltere/third leg imaginal discs (HD) and eye imaginal discs (ED). *Ubx* is expressed in haltere/third leg imaginal discs and is repressed in eye imaginal discs. *so* (*sine oculis*) and *toy* (*twin of eyeless*) both show low expression levels in haltere/third leg imaginal discs and are highly expressed in eye imaginal discs. The ChIP yield (qPCR) of the examined regions was normalized to input DNA and an internal control (*robo3*). Data are expressed as the ratio of ChIP enrichments in haltere/third leg discs versus eye discs. The standard deviation, as indicated by the error bars, was calculated from three independent experiments. At the *Ubx* gene (B–D), a small decrease in the levels of PH was detected in haltere/third leg discs compared to eye discs. Lower levels of PH in haltere/third leg discs correlated with a lower PHO/PHOL ratio. In contrast, slightly higher levels of PH binding were detected in haltere/third leg discs at so and *toy* (E and F), which are repressed in these discs. Higher levels of PH in haltere/third leg discs correlate with a higher PHO/PHOL ratio.

PHO and PHOL share sequence homology, were shown to bind the same DNA motif in vitro, and have been proposed to play redundant roles in PcG-mediated silencing (reviewed in [5]). Notably, we observed that PHO and PHOL binding patterns do not always overlap in the genome. In particular, PHO binds much stronger than PHOL at PH sites (Figures 3, 4, S13C, and S14), whereas both proteins bind with similar intensities in K4-recruiter and K4-TSS clusters (Figures 3 and 4). We also noticed that the majority of PHOL sites in the genome colocalized with TRX-N and H3K4me3-bound regions (Figures 3 and S5; Table S4A). To investigate whether PHO and PHOL may fulfill distinct roles in recruitment of PcG and trxG proteins, we computed the genome-wide ratio of PHO/PHOL binding (see Text S1) and plotted it compared to the individual profiles as well as to PH sites. Figure 7A shows that the PHO/PHOL ratio accurately matches the PH distribution profile since the binding of the two proteins at all other sites in the genome cancels out, whereas PHO binding at PREs is much stronger than PHOL. To confirm whether the ratio of PHO/PHOL is linked to the activity state of PRE/TREs, we examined by quantitative ChIP assays the binding levels of PH, PHO, and PHOL at three PcG target genes characterized by ON/OFF expression states in different larval tissues (Figure 7B–7F). *Ubx* is expressed in haltere/third leg imaginal discs [39] and is repressed in eye imaginal discs (ED). On the contrary, *so* (*sine oculis*) and *toy* (*twin of eyeless*) have very low expression in haltere/third leg discs and are highly expressed in eye discs (Figure S15A). For *Ubx* regulation, we analyzed protein binding levels at the bx PRE, bxd PRE, and the *Ubx* TSS, and for *so* and *toy*, we analyzed their TSS, which overlapped with the PH-bound region (Figure S15B). PH, PHO, and PHOL are bound in all the 5′ regions of the genes that we examined in both the ON and OFF state (Figures 7 and S16). However, significant differences in binding levels were noticed. In haltere/third leg discs where *Ubx* is ON, bx PRE, bxd PRE, and *Ubx* TSS showed a slight decrease in PH binding (50%) as compared to eye discs. Both *so* and *toy* TSS showed higher levels of PH binding in haltere/third leg discs, where these genes are silenced (OFF), as compared to eye imaginal discs (ON). At the *Ubx* TSS and the bx PRE, levels of PHOL were significantly higher in haltere/third leg discs (ON) as compared to eye discs (OFF). With regards to PHO, stronger binding was observed at the PREs in eye discs (OFF state), whereas at *so* and *toy* stronger binding was observed in haltere/third discs (OFF state) compared to eye discs (ON). In summary, a significant decrease in the levels of PH in tissue where target genes are active correlates with a decrease in the PHO/PHOL ratio. On the other hand, increased PH levels at genes that are OFF in a certain tissue correlates well with an increased PHO/PHOL ratio.

**Figure 8:**
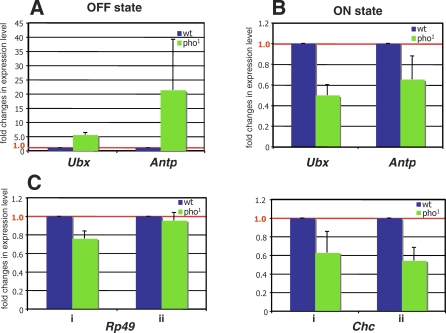
Changes in Transcription Levels of PHO Target Genes in *pho^1^* Mutants Fold changes of *Ubx, Antp*, *Rp49*, and *Chc* expression levels in eye, haltere/third leg and wing imaginal discs in *pho^1^* homozygous mutant larvae (green histograms) compared to wild type (wt; blue histograms). (A) Expression of homeotic genes in eye discs, where both *Ubx* and *Antp* genes are OFF. (B) Expression of homeotic genes in haltere/third leg discs (*Ubx)* and wing discs (*Antp*) where genes are ON. (C) Fold changes in expression levels of *Rp49* and *Chc* in eye (i) and haltere/third leg discs (ii). The standard deviation, as indicated by the error bars, was calculated from at least two independent experiments.

To further examine the function of the PHO/PHOL ratio in Polycomb-dependent gene silencing, we performed quantitative reverse-transcriptase PCR (RT-PCR) on eye, haltere/third leg and wing imaginal discs from wild-type and *pho^1^* homozygous (null mutant allele of PHO [40]) third instar larvae. In wild-type eye discs, the *Ubx* and *Antp* genes are repressed, and the detection of their transcripts is limited to few copies. In *pho^1^* mutant larval eye discs, *Ubx* gene becomes derepressed (5.5-fold), and gene activation is even stronger for the *Antp* gene (between 10- and 30-fold) (Figure 8A). These results suggest that the loss per se of PHO has an impact on the level of transcription of Polycomb-silenced target genes, and this underscores its fundamental role in setting up Polycomb-mediated silencing. Binding of PHOL to the same sequence motif in the promoter region of these two genes might partially complement for the loss of PHO. Indeed, we detected increased binding levels of PHOL to chromatin in *pho^1^* mutant imaginal discs (unpublished data).

We then analyzed the effect of the *pho^1^* mutation in haltere/third leg discs where the *Ubx* gene is transcribed and in wing discs where *Antp* is active. We detected a consistent, yet slight, decrease of their transcripts (2-fold and 1.5-fold, respectively) (Figure 8B). These results suggest that PHO may also play a role as an activator of homeotic genes, even if this role is weaker than its silencing function.

Because we found a high colocalization of PHO and PHOL with TRX-N at many gene promoters not related to PcG-mediated silencing, we performed quantitative RT-PCR to check the expression of two constitutively transcribed genes such as *Chc* and *Rp49*, which are bound by PHO in wild-type embryos. Again, *Chc* expression decreased 1.6 times in both eye and haltere/third leg discs and *Rp49* 1.3 times in eye discs from *pho^1^* mutant larvae (Figure 8C). In contrast, we could not detect major changes in their expression levels in a *phol81A* null mutant background (unpublished data), pointing to a redundant role of PHOL in gene activation

These results, together with the recent work of Beisel et al. [41], indicate that PHO is a modulator, not only of PcG-mediated silencing, but also of the active state of many genes.

## Discussion

The genome-wide mapping of PcG factors, TRX, their associated histone marks, and potential PcG recruiter proteins in *Drosophila* embryos revealed several important features. First, similar to the PcG distribution in *Drosophila* cell lines, PcG proteins strongly colocalize and form large domains containing multiple binding sites. Second, the N-terminal and C-terminal fragments of TRX show different binding affinities to repressed and active chromatin. The N-terminal fragment of TRX has low affinity to PcG binding sites but is strongly bound to thousands of active promoter regions that are trimethylated on H3K4, whereas the C-terminal fragment of TRX only showed high binding affinity to PcG binding sites. Third, the majority of PcG recruiter binding sites are associated with H3K4me3 and TRX-N foci and not with PH binding. The binding ratio between the PHO protein and its homolog PHOL is a major predictive feature of PcG versus TRX recruitment. Finally, supervised and unsupervised sequence analysis methods led to the identification of sequence motifs that discriminate between most of the PcG and TRX binding sites, but these motifs are likely to be working jointly, and none of them seems to drive recruitment by itself.

### Promiscuous Binding Pattern of PcG Recruitment Proteins

To date, PREs have been only characterized in *Drosophila*. These elements are not defined by a conserved sequence, but include several conserved motifs, which are recognized by known DNA binding proteins like GAGA factor (GAF), Pipsqueak (PSQ), Pleiohomeotic and Pleiohomeotic-(like) (PHO and PHOL), dorsal switch protein (DSP1), Zeste, Grainyhead (GH), and SP1/KLF. Our genomic profiles provide a comprehensive view on the potential role of these factors in the establishment of PcG domains.

The presence of PHO at all PREs indicates that PHO is a crucial determinant of PcG-mediated silencing, consistent with earlier analysis on one particular PRE [25,33,42–46]. On the other hand, PHOL and Zeste were bound at a small subset of PREs. Zeste was previously shown to be necessary for maintaining active chromatin states at the *Fab-7* (*Frontabdominal-7*) PRE/TRE [47]. Therefore, Zeste and PHOL may primarily assist transcription rather than PcG-mediated silencing. GAF and DSP1 resemble PHO as they bind to many (albeit less than PHO) PREs as well as to active promoters. Supervised DNA motif analysis indicated a higher density of GAF, DSP1, and PHO binding sites at PREs as compared to other bound regions at non-PH sites. This suggests that cooperative binding of these proteins may provide a platform for PcG protein binding. Moreover, GAF may act by inducing chromatin remodeling [48,49] to remove nucleosomes, since the regions bound by PcG proteins show a characteristic dip in H3K27me3 signal that has been attributed to the absence of nucleosomes in those regions [20,50,51]. These nucleosome depletion sites are the places wherein histone H3 to H3.3 replacement takes place [51]. Indeed, several of the Zeste-bound regions and GAGA binding sequences were shown to localize to peaks of H3.3, suggesting the possibility that GAF may recruit PcG components to PHO-site–containing PREs as well as recruit TRX to promoters via nucleosome disruption.

In addition to an increased density of motifs for GAF, PHO, and PHOL, unsupervised spatial cluster analysis identified specific motifs that distinguish the PH sites from the K4me3 cluster. Although the identity of the factors binding to these motifs is unknown, this suggests that the DNA sequence of PREs contains much of the information needed to recruit PcG proteins and to define silent or active chromatin states. With this distinction, it may be possible to develop an algorithm to faithfully predict the genomic location of PREs. Earlier attempts to predict PREs in the fly genome have made progress toward this goal, but they are still far from reaching the required sensitivity and specificity [19,20,22,24,25] (see also Tables S9 to S11). The use of a sequence analysis pipeline that is not dependent on prior knowledge was demonstrated here to generate new discriminative motifs with a potential predictive power. The unique genomic organization of PcG domains may suggest that the genome is using, not only local sequence (high-affinity transcription factor binding sites located at the binding peaks) information to determine PREs, but also integration of regional sequence information (stronger affinity on 5 kb surrounding PREs). Using such regional information to predict PREs may break the current specificity and sensitivity barriers.

### The PHO versus PHOL Binding Ratio Is a PRE Marker

Our ChIP on chip data showed that PHO binding comes in two distinct flavors. In one class of target sites, PHO binding coincides with PH sites within PC domains, whereas outside these domains, it is largely colocalized with PHOL, TRX-N, and H3K4me3 (Table S4). PHOL binding was weaker at PH sites and was mainly present along with marks associated with gene activation. Quantitative ChIP assays (Figure 7) revealed that PH, PHO, and PHOL were bound in PREs/TSS of their target genes in both ON and OFF states, but the ON state was marked by a decrease in PH binding and a corresponding increase in PHOL levels, whereas the OFF state was characterized by an increase in both PH and PHO binding levels.

Papp and Muller [39] analyzed chromatin at the *Ubx* TSS, the bx PRE, and the bxd PRE (the same primers were used in our study) by comparing haltere/third leg imaginal discs (ON state) with wing imaginal discs (OFF state). They found a 50% reduction of PH binding levels at the bx PRE, a minor decrease at bxd, and no change in the *Ubx* TSS. Our ChIP experiments demonstrated a 50% decrease in PH levels at bx PRE and at the *Ubx* TSS and a minor decrease at bxd PRE when comparing haltere/third leg imaginal discs to eye imaginal discs. We also observed a slight decrease in the levels of PHO in haltere/third leg disc (ON state) as compared to eye imaginal discs (OFF state) at the bx and bxd PRE, whereas Papp and Muller [39] did not see differences in the levels of PHO. The most likely explanation for these discrepancies is that the peripodal membrane cells of the wing imaginal discs express *Ubx*, whereas all cells silence this gene in eye imaginal discs.

In *pho^1^* mutant eye discs, the absence of PHO causes derepression of the homeotic genes *Ubx* and *Antp*. However, the expression levels in *pho^1^* mutants are still much weaker compared to tissues where these genes are normally expressed. This low degree of activation could be explained by compensatory binding of PHOL to the PHO sites in order to maintain PcG-mediated silencing, even if the PHOL-dependent rescue function is incomplete as *pho^1^* mutants die as pharate adults. PHO and PHOL have indeed been described as redundant in their role in PcG-mediated silencing since they bind to the same DNA sequence motif in vitro. However, out of the 1,757 places wherein both PHO and PHOL were significantly bound, only 807 shared the same local maxima (46%). Another 559 (32%) peaks were within 250 bp of each other. This suggests that, in vivo, these two proteins prefer slightly different sequences, with PHO more strongly attracted to PREs, whereas PHOL binds better to promoters. Moreover, PHO interacts directly with PC and PH [13], as well as with the PRC2 components E(z) and Esc, whereas PHOL only interacts with Esc in yeast two-hybrid assays [12]. Stronger interactions between PHO and PcG components may stabilize PHO binding at PREs, favoring it over the binding of PHOL. It is thus possible that the primary function of PHOL is as a transcription cofactor, and that its recruitment to PREs is subsidiary to PHO.

### The Double Life of TRX

Here, we report for the first time, to our knowledge, the genome-wide distribution of TRX. This protein has been proposed to counteract PcG-mediated silencing [52]. Petruk et al. [53] demonstrated that TRX colocalizes with Polymerase II and elongation factors in *Drosophila* polytene chromosomes. They then showed that PcG and TRX proteins bind to a PRE mutually exclusively in salivary gland chromosomes [54]. In contrast, two other studies [39,41] found binding of TRX at discrete sites at PREs and promoter regions of HOX genes, and suggested that TRX coexists with PRC1 components at silent genes. We postulated that these differences might be explained by the use of different TRX antibodies, one against the N-terminal domain [53] and one against the C-terminal domain of TRX [39,41]. Notably, the TRX protein is proteolytically cleaved into an N-terminal and a C-terminal domain [10], but the fate of the two moieties after cleavage has never been addressed in vivo.

Our genome-wide mapping studies using the same antibody against the N-terminal fragment (TRX-N) as used by Petruk et al. [53], showed that the binding affinity of the N-terminal fragment to PREs is rather weak, whereas TRX-N binds thousands of promoter regions trimethylated on H3K4, indicating a general role of TRX-N in gene activation. In contrast, ChIP on chip profiling using an antibody against the C-terminal TRX fragment showed high binding levels at PRE/TREs, whereas binding to promoter regions (where the TRX N-terminal fragment is strongly bound) is rather weak. The strong quantitative correlation between the binding intensities of PH and TRX-C suggests that TRX-C can indeed bind to silent PcG target genes. These data are confirmed by the colocalization of PH and TRX-C at inactive Hox genes in salivary gland polytene chromosomes and in diploid cell nuclei (as seen in a combination of DNA fluorescent in situ hybridization (FISH) and immunostaining; unpublished data). Thus, PcG silencing may involve locking the C-terminal portion of TRX in an inactive state that perturbs transcription activation events. The fact that TRX is recognized by two different antibodies that recognize PREs (H3K4me3-depleted regions) or TSSs suggests that these antibodies reflect the activity state of the protein and thus represent a powerful tool to study the switching of genes between silencing and activation.

### Plasticity of Polycomb Binding Profiles in *Drosophila* Embryos versus *Drosophila* Cell Lines

Similar to mapping studies in *Drosophila* cell lines, H3K27me3 also forms large domains in *Drosophila* embryos. These large PcG domains could provide the basis of a robust epigenetic memory to maintain gene expression states during mitosis. As previously suggested [55], stably bound PcG complexes at PREs may loop out and form transient contacts with neighboring chromatin, which become trimethylated on H3K27. H3K27me3 might then attract the chromodomain of the PC protein, which may be occasionally trapped at these remote sites by cross-linking mediated by the chromodomain of PC. Alternatively, PcG subcomplexes missing some of the subunits might spread from the PRE into flanking genomic regions containing H3K27me3 histones.

Although genome-wide PcG profiles in *Drosophila* embryos correlate well with profiles from *Drosophila* cell lines, it has recently been shown that PcG protein binding profiles are partially remodeled during development [19,30]. Comparison of our PcG target genes (Figure S19 and Tables S14–S16) with Schwartz et al. [20] showed that 40% of our targets were unique (Figure S17). The fact that a consistent number of targets are only found in one or two of the samples indicates tissue specific PcG occupancy. Thus, although PcG proteins have been often invoked as epigenetic gatekeepers of cellular memory processes, they may be involved as well in dynamic gene regulation during fly development [19,56], similar to their function in mammalian cells.

## Materials and Methods

### Antibodies.

All antibodies used in this study are listed in Table S12.

### ChIP on chip experiments on whole *Drosophila* embryos.

ChIP assays were performed on 4–12-h-old embryos of the Oregon-R *w1118* line of Drosophila melanogaster. The complete experimental details of the ChIP experiments are available in Text S1. Briefly, ChIP samples were amplified by ligation-mediated (LM) PCR, as described previously [19], and hybridized to whole-genome tiling arrays manufactured by NimbleGen Systems (the array design is described in Text S1). A list of all significantly enriched regions (*p*-value < 0.0001) for all profiles are shown in Table S17.

### Spatial clustering and motif analysis.

Spatial clustering was performed by training a Hidden Markov Model (HMM) to fit the available genomic profiles using a small set of clusters. The HMM represents both the relations between clusters and the joint profile distribution emitted from each cluster. We developed a hierarchical version of the algorithm so that the two layers of genomic organization in the data can be characterized (for details, see Text S1). We further enhanced the spatial clustering framework to search for motifs that discriminate among clusters. We also used the MEME and Motif Alignment and Search Tool (MAST) programs to search for enriched motifs directly [57,58] (a detailed description can be found in Text S1).

### ChIP analysis of *Drosophila* imaginal discs using quantitative PCR analysis.

ChIP assays of imaginal discs were performed as described for embryos with the following modifications: third instar larval eye discs and haltere/third leg discs were dissected in SS M3 insect medium and kept on ice during dissection. A hundred discs were used per immunoprecipitation (IP). Discs were pelleted by centrifugation at 4.000 *g* for 5 min, resuspended in 1 ml of Buffer A1, and then cross-linked for 15 min in the presence of 1.8% formaldehyde by homogenization in a Tenbroeck homogenizer. Chromatin was sonicated using a Bioruptor (Diagenode) for 12 min (settings 30 s on, 30 s off, high power). Sheared chromatin had an average length of 500 to 1,000 bp. Antibodies used for IP (PHO, PHOL, and PH) were diluted 1:100 (PH and PHO) or 1:20 (PHOL). Enrichment of specific DNA fragments was analyzed by real-time PCR, using Roche Light Cycler equipment and accessories as described in Comet et al. [59]. Enrichment in specific IPs was determined by normalizing the amount of DNA obtained in each reaction by the amount of a negative control fragment from the *robo3* gene. Primer sequences are listed in Table S13.

### RT PCR of *pho^1^* imaginal discs.

*pho^1^* homozygous larvae were collected from a stock ey-GAL4/ey-GAL4; pho1/GS15194 kindly provided by R. Paro's lab [41]. Wild-type and *pho^1^/pho^1^* mutant larvae were dissected in PBS, and 40 eye or haltere/third leg discs were taken for RNA isolation using TRIzol reagent (Invitrogen). RT-PCR was performed using Superscript III First Strand Synthesis Kit from Invitrogen following the manufacturer's instructions. Reverse transcription was primed using hexamer primers. Quantitative polymerase chain reaction (qPCR) analysis was done as described for ChIP experiments. The copy number for each investigated gene was normalized to the copy number of the 18S RNA gene. Primer sequences are listed in Table S13.

### Accession numbers.

Experiment, first part (combined replicates; K27, PC, PH, PHO, DSP1, PHOL, GAF, TRX-N, and K4): E-MEXP-1708.

Additionally, all employed microarray designs have their own accessions: PhysicalArrayDesign name: 2005-08-08_Henikoff_Dros_ChIP_1, ArrayExpress accession: A-MEXP-1251; PhysicalArrayDesign name: 2005-08-08_Henikoff_Dros_ChIP_2, ArrayExpress accession: A-MEXP-1252; PhysicalArrayDesign name: 2005-08-08_Henikoff_Dros_ChIP_3, ArrayExpress accession: A-MEXP-1253; PhysicalArrayDesign name: 2007-03-13_Henikoff_Dros_ChIP_1, ArrayExpress accession: A-MEXP-1254; PhysicalArrayDesign name: 2007-03-13_Henikoff_Dros_ChIP_2, ArrayExpress accession: A-MEXP-1255; PhysicalArrayDesign name: 2007-03-13_Henikoff_Dros_ChIP_3, ArrayExpress accession: A-MEXP-1256; and PhysicalArrayDesign name: Cavalli_Dmel_1_tiling, ArrayExpress accession: A-MEXP-1257.

Gene accession numbers: *Antp*: FBgn0000095; *ato*: FBgn0010433; *cad*: FBgn0000251; *Chc*: FBgn0000319; *Dll*: FBgn0000157; *dsx*: FBgn0000504; *grn*: FBgn0001138; *hb*: FBgn0001180; *robo3*: FBgn0041097; *Rp49*: FBgn0002626; *so*: FBgn0003460; *toy*: FBgn0019650; and *ubx*: FBgn0003944.

## Supporting Information

Figure S1 Quality Control of Biological ChIP on Chip Replicates Plots showing correlation between normalized log_2_ ratio of replicate 1 versus replicate 2 for each profile. Probes having a statistically significant log_2_ ratio (combined *p*-value < 0.0001) are highlighted in red. The significant probes show good correlation between biological replicates.
(482 KB PPT)

Figure S2 Quality Control of Biological ChIP on Chip Replicates In each pair of rows, the upper panel shows correlation plots between replicate 1 versus replicate 2 mock (green) signal intensities, whereas the lower panel shows correlation plots between replicate 1 versus replicate 2–specific IP (dark red) signal intensities for each chromatin profile. Probes having a statistically significant log_2_ ratio (combined *p*-value < 0.0001) are highlighted in red.
(1.35 MB PPT)

Figure S3 Histograms Representing the Size Distribution of ChIP on Chip Bound Regions Only profiles that showed localized binding (and PC) were analyzed. All the bound regions were with *p*-value < 1E−04. Note that in all the profiles except H3K4me3, the majority of bound regions were of length less than 2,000 bp.
(968 KB PPT)

Figure S4 Size, Number of PH Binding Sites, and Number of Genes within H3K27me3 Domains (A) Size distribution of H3K27me3 domains.
(B) Distribution of PH peaks in H3K27me3 domains. The majority of domains had at least one PH peak. The largest number of PH peaks (30) was found in Hox cluster (CHR3R:12,482,959–12,811,306 bp). Twenty-two PH peaks were not present within H3K27me3 domains. Here, PH peaks denote those that are present along with PC.
(C) Distribution of genes in H3K27me3 domains. The majority of domains have at least one gene. The largest number of genes was present in a domain in chromosome 3L (1,338,575–1,457,527 bp): 18 genes.
(339 KB PPT)

Figure S5 Overview of Global Correlations between All the Profiles Whose Genome-Wide Binding Was Determined by ChIP on Chip (A) Probe by probe correlation of the log_2_ ratios was done for each pair of profiles. K27 denotes H3K27me3, and K4 denotes H3K4me3.
(B) Table indicating percentage of the genome covered by each single protein or histone modification.
(138 KB PPT)

Figure S6 Probe-Wise Correlation between Profiles Green and red denote the significantly enriched probes, and grey denotes the nonsignificant ones. Common significantly enriched probes of *x-* and *y*-axis profiles are shown in orange. Note that almost all the probes bound with PH also are enriched for PC and PHO.
(6.15 MB PPT)

Figure S7 Illustration of the Clustering Method Plots showing ChIP on chip profiles aligned with the different spatial clusters (modes) along part of chromosome 3R. ChIP on chip plots show the ratios (fold change) of specific IP versus mock IP. Significantly enriched fragments (*p*-value < 1E−04) are shown in red. Gray bars in the “Modes profiles” indicate posterior probabilities for the association of probes with a cluster.
(506 KB PPT)

Figure S8 Overrepresented DNA Motifs in ChIP on Chip Bound Regions of PH Sites with PcG Recruitment Factors and Sites Without PH but With PcG Recruitment Factors The “motif length” parameter is 5–10 bp. The MEME E-value of each motif is shown beside its name.
(A) Overrepresented DNA motifs at PH- and PC-bound regions with PHO, DSP1, and GAF.
(B) Overrepresented DNA motifs at regions bound with PHO, DSP1, and GAF (absence of PH and PC).
(1.25 MB PPT)

Figure S9 Overrepresented Motifs at DSP1 Binding Sites (A) Overrepresented motifs in the complete set of DSP1-bound regions (E-value 6.8e−868). A 300-bp region around the Lmax was taken out for searching motifs. Zoops model and 5–10-bp motif width parameters were used.
(B) Overrepresented motifs in DSP1-bound regions wherein GAF binding is not detected (E-value 1.4e−013). A total of 41/77 bound regions contained this motif.
(341 KB PPT)

Figure S10 Overrepresented Motifs at PHO and PHOL Binding Sites (A) Overrepresented motifs in PHO-bound regions with no detectable PHOL (479), E-value 1.8e−100 (set1).
(B) Overrepresented motifs in PHOL-bound regions with no detectable PHO (30), E value 2.2e-003 (set2).
(330 KB PPT)

Figure S11 Overrepresented Sequence Motifs at the 441 PH Binding Sites (A) A 300-bp sequence around the local maxima of intensity of a PH-bound region was analyzed for sequence motifs.
(B) A 500-bp region around the local maxima of intensity was analyzed. Zoops model in MEME was used and the E-value of each motif is shown beside its name. Both sequence sets yielded the same overrepresented motifs. Note the absence of PHO motif in the enriched motifs list.
(1.42 MB PPT)

Figure S12 Average intensity of recruitment factors in PH- and non-PH–bound regions Average intensities for GAF, DSP1, and PHO, but not PHOL, are higher at PH binding sites as compared to non-PH–bound regions.
(77 KB PPT)

Figure S13 Validation of ChIP on Chip Results by qPCR ChIP assays were performed on 4–12-h-old whole *Drosophila* embryos. Before amplification and hybridization on microarrays, specific enrichments of several regions were quantified by qPCR. Three regions that were known to be bound by PcG proteins (bxd PRE, *Dll*, and *cad*) and two control regions (*Rp49* and *robo3*) were analyzed.
(A) ChIP assays with PC, PH, H3K27me3, H3K4me3, and TRX antibodies. Copy number of the PCR fragments enriched in the ChIP experiments are represented for each region analyzed.
(B) ChIP assays with PcG recruitment factors PHO, PHOL, DSP1, and GAF. Copy number of the PCR fragments enriched in the ChIP experiment are represented for each region analyzed.
(C). ChIP assays of PHO and PHOL replotted side by side for better comparison. Note that higher levels of PHO and very low levels of PHOL are seen in PH-bound regions, whereas higher levels of PHOL are seen at the Rp49 promoter. The *robo3* amplicon is located in the coding region of the gene; hence, enrichment of all examined proteins is low.
(86 KB PPT)

Figure S14 PHO- and PHOL-Binding Ratio at PH-Bound Sites (A) Average intensity ratio of PHO/PHOL in PH-bound sites {PHO OR PHOL AND PH} and non-PH–bound regions {PHO OR PHOL AND NOT PH}.
(B) Average intensity of PHO in PH-bound {PHO OR PHOL AND PH} and non-PH–bound {PHO OR PHOL AND NOT PH} regions.
(C) Average intensity of PHOL in PH-bound {PHO OR PHOL AND PH} and non-PH–bound {PHO OR PHOL AND NOT PH} regions. In PH and non-PH regions, the first significant peak of PHO/PHOL is looked for. If a significant peak is present for one of them, then the intensity of the other, even if it not significant, is recorded, and a ratio between PHO/PHOL is calculated.
(75 KB PPT)

Figure S15 Expression Status (Eye versus Haltere/Third Leg Imaginal Discs.) and Chromatin Profiles (Embryos) of *Ubx*, *so* (*sine oculis*), and *toy* (*twin of eyeless*) (A) Expression status of *so* and *toy* in eye and haltere/third leg imaginal discs. The cDNA copy number was quantified using qPCR. Note that both the genes are highly expressed in eye imaginal discs, whereas their expression levels are low (*so*) or not detectable (*toy*) in haltere/third leg discs.
(B) ChIP on chip profile in *Drosophila* embryos of PcG proteins, PHO, PHOL, and H3K27me3 at the *Ubx*, *so* (*sine oculis*), and *toy* (*twin of eyeless*) genes.
(178 KB PPT)

Figure S16 Binding of PH, PHO, and PHOL at PcG Target Genes in ON and OFF States Same experiment as shown in Figure 7. ChIP enrichment at PREs/TSS of PcG target genes for PH, PHO, and PHOL antibodies in haltere/third leg imaginal discs (HD) and eye imaginal discs (ED). The data are expressed as the percentage of input chromatin precipitated for each region examined. The mean values ± standard deviations of three independent ChIP experiments are shown.
(109 KB PPT)

Figure S17 Comparison of PcG Target Genes with Other Published Genome-Wide Datasets A total of 63.79% of our target genes overlapped with Schwartz et al. [20] (S2 cell line). Schwartz et al. [20] defined strong PcG sites as those that showed simultaneous strong binding of PC, PSC, E(Z), and H3K27me3 (above 2-fold enrichment). A total of 188 genes from these regions that showed both PcG binding and methylation were defined as strong PcG targets. Weak PcG sites were defined as those wherein binding for one of the profiles (PC, PSC, E(Z), and H3K27me3) was lower and below the threshold levels. Seventy-four target genes were assigned to these regions. We separately compared our list of target genes to strong and weak PcG targets of Schwartz et al. [20]: 137/188 (73%) of strong target genes and 18/74 (24.3%) of the weak target genes of Schwartz et al. [20] matched our list. The majority of the strong targets are present in our list, showing that significant binding of multiple PcG proteins might be indicating genuine PcG targets. A total of 13.17% of our target genes were predicted by Ringrose et al. [25]; 27.57% of our target genes overlapped with Tolhuis et al. [22], but these authors only analyzed 30% of the genome using the DamID technique (unpublished data).
(85 KB PPT)

Figure S18 The Spatial Cluster Model Is Defined Based on a Set of Clusters and an HMM Structure Imposed over Them Each cluster represents a combinatorial pattern among transcription factor (TF) occupancies and histone mark densities (as shown in Figure 3). The HMM structure defines the probability of observing each of the clusters given the cluster covering the previous genomic locus. Shown here are the spatial cluster model HMM states for the PcG/trxG model and the main transitions (conditional probabilities larger than 5% and 1%) in the model. Arc widths schematically reflect transition probability. The TSS enrichment (as in Figure 3) is provided for reference. Note that although the model is defined as directional, we always train it using the forward strand direction, so it lacks real “directionality” as expected from transcriptional units. The figure shows directional edges since the transition probability is always relative to the general cluster frequency, so transitions from very common states (e.g., background states) are occurring often but have low conditional probability, whereas transitions from rare states (e.g., PREs) occurs with high conditional probability.
(285 KB PPT)

Figure S19 Functional Characterization of PcG Targets The PcG target genes were functionally categorized using the Gene Ontology (GO) toolbox [60]. The “molecular function” ontology, the hypergeometric statistical test and Benjamini and Hochberg correction for multiple testing parameters were used for the classification. The whole genome was used as the reference set. Only the significantly enriched or depleted classes are shown.
(2.02 MB PPT)

Table S1 Number of Significantly Bound Regions for Each Protein Profile The data are shown for two different *p*-value cutoffs.
(14 KB XLS)

Table S2 Lists of PcG Target Genes (A) Target genes for PH peaks present in PC- and H3K27me3-bound regions.
(B) Target genes corresponding to PH peaks within PC-bound region but with weak H3K27me3 that does not cross the *p*-value threshold.
(23 KB XLS)

Table S3 Combination of Recruiters in the PH Sites as Compared to Their Occurrence in the Genome (15 KB XLS)

Table S4 Distribution of PcG Recruiters in the Genome (A) Various combinations of PcG recruitment factors present in the genome. Note that PHO and PHOL do not colocalize at all the regions. However, very few regions are present in the genome where we could see PHOL sites without PHO. At the genome-wide level, DSP1 largely colocalizes with GAF and PHO.
(B) Various combinations of PcG recruitment factor binding sites, H3K4me3, and TRX-N in the genome.
(C) Various combinations of Zeste-bound regions in the genome with the other PcG recruiters, TRX-N and H3K4me3. P*, PL*, K4*, and Z denote PHO, PHOL, H3K4me3, and Zeste, respectively. The hash mark (#) denotes the number of regions. Zeste ChIP on chip data were taken from [31]
(D) Various combinations of TRX-C, TRX-N and H3K4me3 in the genome.
(25 KB XLS)

Table S5 Number of Sequences with Motifs The patser program was used for this analysis. The position-specific probability matrix (PSPM) of the MEME motifs (motif width 5–10 bp) were taken as input for patser. The motifs were counted in PH and non-PH regions bound with recruitment factors (PHO+DSP1+GAF). ‘NC' denotes not calculated. The density of motif in each sequence set was also calculated. The total number of base pairs in each sequence set was calculated after concatenating the entire sequence into a single string. In PRE regions, am1, am3, and bm3 were present in one motif per 168 bp, 66 bp, and 2,464 bp, whereas in non-PRE regions, the same motifs were present at one motif per 356 bp, 112 bp, and 9,047 bp, respectively.
(16 KB XLS)

Table S6 Specific Enrichment of Motifs in ChIP on Chip Bound Regions. MEME top motif (default “motif width” parameter) sequences used in MEME along with two control sets were taken as input. Control 1 denotes random regions wherein none of our tested proteins/histone modifications showed binding. Control 2 denotes random regions from the genome (Materials and Methods). The data reveal the specific enrichment of each motif in ChIP on chip bound regions. The MAST program was used for analysis.
(15 KB XLS)

Table S7 Number of ChIP on Chip Bound Sequences with Motifs The patser program was used for this analysis. The position-specific scoring matrix (PSSM) of the top MEME motif (default “motif width”) was taken as input for patser. The motif was counted in three sets of sequences: Set1: sequences around the Lmax of each ChIP on chip bound region (column 2); Set2: the complete sequence of the bound region (column 3); and Set3: the input sequences taken for MEME (column 4).
(15 KB XLS)

Table S8 Frequency of MEME Motifs in PcG Recruitment Factor–Bound Regions With PH and Without PH PHO, DSP1, and GAF motifs had higher frequency in PH-bound regions as compared to other places wherein they were bound without PH. A *t*-test was done to look for the difference in distribution of motif frequency between recruitment factor–bound regions with PH and without PH.
(14 KB XLS)

Table S9 Comparison of PREdictor Predictions with Our ChIP on Chip Data A total of 53/344 (15.41%) predicted regions showed PH + PC binding in ChIP on chip; 53/439 (12.07%) of ChIP on chip PH+PC-bound regions were predicted by PREdictor. The predictions of chromosome 3R were marginally more validated in our data as compared to other chromosomes. This could be because the majority of the input PRE regions for PREdictor were taken from chromosome 3R.
(14 KB XLS)

Table S10 Comparison of Predictions with Different Combinations of ChIP on Chip Bound Regions, Especially of PcG Recruitment Factors All the bound sites had *p*-value < 1E−04 for each factor.
(14 KB XLS)

Table S11 Correlation between PREdictor Score and PH+PC Occupancy Detected by ChIP on Chip All the bound sites had *p*-value < 1E-04 for each factor.
(15 KB XLS)

Table S12 Details of the Antibodies Used for the Chromatin Immunoprecipitation Assays (16 KB XLS)

Table S13 Details of Primers Used in the Study (15 KB XLS)

Table S14 GO Classification of PcG Target Genes with “Molecular Function” Ontology (56 KB XLS)

Table S15 GO Classification of PcG Target Genes with “Biological Process” Ontology (254 KB XLS)

Table S16 GO Classification of PcG Target Genes with “Cellular Component” Ontology (43 KB XLS)

Table S17 Lists of ChIP on Chip Enriched Regions (*p-*Value < 0.0001) (1.04 MB XLS)

Text S1 Supporting Information. This text includes supporting results and discussion, and detailed materials and methods. (148 KB DOC)

## Abbreviations

ChIP: chromatin immunoprecipitation
HMM: Hidden Markov Model
IP: immunoprecipitation
MEME: Multiple EM for motif Elicitation
PcG: Polycomb group
PRE: polycomb group response element
qPCR: quantitative polymerase chain reaction
TRE: trithorax group response element
trxG: trithorax group
TSS: transcription start site

## References

[pbio-1000013-b001] Schwartz YB, Pirrotta V (2008). Polycomb complexes and epigenetic states. Curr Opin Cell Biol.

[pbio-1000013-b002] Sparmann A, van Lohuizen M (2006). Polycomb silencers control cell fate, development and cancer. Nat Rev Cancer.

[pbio-1000013-b003] Schwartz YB, Pirrotta V (2007). Polycomb silencing mechanisms and the management of genomic programmes. Nat Rev Genet.

[pbio-1000013-b004] Muller J, Kassis JA (2006). Polycomb response elements and targeting of Polycomb group proteins in Drosophila. Curr Opin Genet Dev.

[pbio-1000013-b005] Schuettengruber B, Chourrout D, Vervoort M, Leblanc B, Cavalli G (2007). Genome regulation by polycomb and trithorax proteins. Cell.

[pbio-1000013-b006] Cao R, Zhang Y (2004). The functions of E(Z)/EZH2-mediated methylation of lysine 27 in histone H3. Curr Opin Genet Dev.

[pbio-1000013-b007] Saurin AJ, Shao Z, Erdjument-Bromage H, Tempst P, Kingston RE (2001). A Drosophila Polycomb group complex includes Zeste and dTAFII proteins. Nature.

[pbio-1000013-b008] Grimaud C, Negre N, Cavalli G (2006). From genetics to epigenetics: the tale of Polycomb group and trithorax group genes. Chromosome Res.

[pbio-1000013-b009] Hsieh JJ, Cheng EH, Korsmeyer SJ (2003). Taspase1: a threonine aspartase required for cleavage of MLL and proper HOX gene expression. Cell.

[pbio-1000013-b010] Hsieh JJ, Ernst P, Erdjument-Bromage H, Tempst P, Korsmeyer SJ (2003). Proteolytic cleavage of MLL generates a complex of N- and C-terminal fragments that confers protein stability and subnuclear localization. Mol Cell Biol.

[pbio-1000013-b011] Otte AP, Kwaks TH (2003). Gene repression by Polycomb group protein complexes: a distinct complex for every occasion. Curr Opin Genet Dev.

[pbio-1000013-b012] Wang L, Brown JL, Cao R, Zhang Y, Kassis JA (2004). Hierarchical recruitment of polycomb group silencing complexes. Mol Cell.

[pbio-1000013-b013] Mohd-Sarip A, Venturini F, Chalkley GE, Verrijzer CP (2002). Pleiohomeotic can link polycomb to DNA and mediate transcriptional repression. Mol Cell Biol.

[pbio-1000013-b014] Dejardin J, Rappailles A, Cuvier O, Grimaud C, Decoville M (2005). Recruitment of Drosophila Polycomb group proteins to chromatin by DSP1. Nature.

[pbio-1000013-b015] Brown JL, Fritsch C, Mueller J, Kassis JA (2003). The Drosophila pho-like gene encodes a YY1-related DNA binding protein that is redundant with pleiohomeotic in homeotic gene silencing. Development.

[pbio-1000013-b016] Boyer LA, Plath K, Zeitlinger J, Brambrink T, Medeiros LA (2006). Polycomb complexes repress developmental regulators in murine embryonic stem cells. Nature.

[pbio-1000013-b017] Bracken AP, Dietrich N, Pasini D, Hansen KH, Helin K (2006). Genome-wide mapping of Polycomb target genes unravels their roles in cell fate transitions. Genes Dev.

[pbio-1000013-b018] Lee TI, Jenner RG, Boyer LA, Guenther MG, Levine SS (2006). Control of developmental regulators by Polycomb in human embryonic stem cells. Cell.

[pbio-1000013-b019] Negre N, Hennetin J, Sun LV, Lavrov S, Bellis M (2006). Chromosomal distribution of PcG proteins during Drosophila development. PLoS Biol.

[pbio-1000013-b020] Schwartz YB, Kahn TG, Nix DA, Li XY, Bourgon R (2006). Genome-wide analysis of Polycomb targets in Drosophila melanogaster. Nat Genet.

[pbio-1000013-b021] Squazzo SL, O'Geen H, Komashko VM, Krig SR, Jin VX (2006). Suz12 binds to silenced regions of the genome in a cell-type-specific manner. Genome Res.

[pbio-1000013-b022] Tolhuis B, de Wit E, Muijrers I, Teunissen H, Talhout W (2006). Genome-wide profiling of PRC1 and PRC2 Polycomb chromatin binding in Drosophila melanogaster. Nat Genet.

[pbio-1000013-b023] Manak JR, Dike S, Sementchenko V, Kapranov P, Biemar F (2006). Biological function of unannotated transcription during the early development of Drosophila melanogaster. Nat Genet.

[pbio-1000013-b024] Fiedler T, Rehmsmeier M (2006). jPREdictor: a versatile tool for the prediction of cis-regulatory elements. Nucleic Acids Res.

[pbio-1000013-b025] Ringrose L, Rehmsmeier M, Dura JM, Paro R (2003). Genome-wide prediction of Polycomb/Trithorax response elements in Drosophila melanogaster. Dev Cell.

[pbio-1000013-b026] Bernstein BE, Mikkelsen TS, Xie X, Kamal M, Huebert DJ (2006). A bivalent chromatin structure marks key developmental genes in embryonic stem cells. Cell.

[pbio-1000013-b027] Mikkelsen TS, Ku M, Jaffe DB, Issac B, Lieberman E (2007). Genome-wide maps of chromatin state in pluripotent and lineage-committed cells. Nature.

[pbio-1000013-b028] Pan G, Tian S, Nie J, Yang C, Ruotti V (2007). Whole-genome analysis of histone H3 lysine 4 and lysine 27 methylation in human embryonic stem cells. Cell Stem Cell.

[pbio-1000013-b029] Zhao XD, Han X, Chew JL, Liu J, Chiu KP (2007). Whole-genome mapping of histone H3 Lys4 and 27 trimethylations reveals distinct genomic compartments in human embryonic stem cells. Cell Stem Cell.

[pbio-1000013-b030] Kwong C, Adryan B, Bell I, Meadows L, Russell S (2008). Stability and dynamics of polycomb target sites in Drosophila development. PLoS Genet.

[pbio-1000013-b031] Moses AM, Pollard DA, Nix DA, Iyer VN, Li XY (2006). Large-scale turnover of functional transcription factor binding sites in Drosophila. PLoS Comput Biol.

[pbio-1000013-b032] Biggin MD, Tjian R (1988). Transcription factors that activate the Ultrabithorax promoter in developmentally staged extracts. Cell.

[pbio-1000013-b033] Brown JL, Mucci D, Whiteley M, Dirksen ML, Kassis JA (1998). The Drosophila Polycomb group gene pleiohomeotic encodes a DNA binding protein with homology to the transcription factor YY1. Mol Cell.

[pbio-1000013-b034] Kim LK, Choi UY, Cho HS, Lee JS, Lee WB (2007). Down-regulation of NF-kappaB target genes by the AP-1 and STAT complex during the innate immune response in Drosophila. PLoS Biol.

[pbio-1000013-b035] Tanay A (2006). Extensive low-affinity transcriptional interactions in the yeast genome. Genome Res.

[pbio-1000013-b036] Orian A, van Steensel B, Delrow J, Bussemaker HJ, Li L (2003). Genomic binding by the Drosophila Myc, Max, Mad/Mnt transcription factor network. Genes Dev.

[pbio-1000013-b037] Pi H, Huang SK, Tang CY, Sun YH, Chien CT (2004). phyllopod is a target gene of proneural proteins in Drosophila external sensory organ development. Proc Natl Acad Sci U S A.

[pbio-1000013-b038] Brown JL, Grau DJ, DeVido SK, Kassis JA (2005). An Sp1/KLF binding site is important for the activity of a Polycomb group response element from the Drosophila engrailed gene. Nucleic Acids Res.

[pbio-1000013-b039] Papp B, Muller J (2006). Histone trimethylation and the maintenance of transcriptional ON and OFF states by trxG and PcG proteins. Genes Dev.

[pbio-1000013-b040] Klymenko T, Papp B, Fischle W, Kocher T, Schelder M (2006). A Polycomb group protein complex with sequence-specific DNA-binding and selective methyl-lysine-binding activities. Genes Dev.

[pbio-1000013-b041] Beisel C, Buness A, Roustan-Espinosa IM, Koch B, Schmitt S (2007). Comparing active and repressed expression states of genes controlled by the Polycomb/Trithorax group proteins. Proc Natl Acad Sci U S A.

[pbio-1000013-b042] Mihaly J, Mishra RK, Karch F (1998). A conserved sequence motif in Polycomb-response elements. Mol Cell.

[pbio-1000013-b043] Fritsch C, Brown JL, Kassis JA, Muller J (1999). The DNA-binding polycomb group protein pleiohomeotic mediates silencing of a Drosophila homeotic gene. Development.

[pbio-1000013-b044] Shimell MJ, Peterson AJ, Burr J, Simon JA, O'Connor MB (2000). Functional analysis of repressor binding sites in the iab-2 regulatory region of the abdominal-A homeotic gene. Dev Biol.

[pbio-1000013-b045] Busturia A, Lloyd A, Bejarano F, Zavortink M, Xin H (2001). The MCP silencer of the Drosophila Abd-B gene requires both Pleiohomeotic and GAGA factor for the maintenance of repression. Development.

[pbio-1000013-b046] Mishra RK, Mihaly J, Barges S, Spierer A, Karch F (2001). The iab-7 polycomb response element maps to a nucleosome-free region of chromatin and requires both GAGA and pleiohomeotic for silencing activity. Mol Cell Biol.

[pbio-1000013-b047] Dejardin J, Cavalli G (2004). Chromatin inheritance upon Zeste-mediated Brahma recruitment at a minimal cellular memory module. Embo J.

[pbio-1000013-b048] Orphanides G, LeRoy G, Chang CH, Luse DS, Reinberg D (1998). FACT, a factor that facilitates transcript elongation through nucleosomes. Cell.

[pbio-1000013-b049] Orphanides G, Wu WH, Lane WS, Hampsey M, Reinberg D (1999). The chromatin-specific transcription elongation factor FACT comprises human SPT16 and SSRP1 proteins. Nature.

[pbio-1000013-b050] Mohd-Sarip A, van der Knaap JA, Wyman C, Kanaar R, Schedl P (2006). Architecture of a polycomb nucleoprotein complex. Mol Cell.

[pbio-1000013-b051] Mito Y, Henikoff JG, Henikoff S (2007). Histone replacement marks the boundaries of cis-regulatory domains. Science.

[pbio-1000013-b052] Ingham PW (1983). Differential expression of bithorax complex genes in absence of the *extra sex combs* and *trithorax* genes. Nature.

[pbio-1000013-b053] Petruk S, Sedkov Y, Riley KM, Hodgson J, Schweisguth F (2006). Transcription of bxd noncoding RNAs promoted by trithorax represses Ubx in cis by transcriptional interference. Cell.

[pbio-1000013-b054] Petruk S, Smith ST, Sedkov Y, Mazo A (2008). Association of trxG and PcG proteins with the bxd maintenance element depends on transcriptional activity. Development.

[pbio-1000013-b055] Kahn TG, Schwartz YB, Dellino GI, Pirrotta V (2006). Polycomb complexes and the propagation of the methylation mark at the Drosophila ubx gene. J Biol Chem.

[pbio-1000013-b056] Oktaba K, Gutierrez L, Gagneur J, Girardot C, Sengupta AK (2008). Dynamic regulation by polycomb group protein complexes controls pattern formation and the cell cycle in Drosophila. Dev Cell.

[pbio-1000013-b057] Bailey TL, Elkan C (1994). Fitting a mixture model by expectation maximization to discover motifs in biopolymers. Proc Int Conf Intell Syst Mol Biol.

[pbio-1000013-b058] Bailey TL, Gribskov M (1998). Methods and statistics for combining motif match scores. J Comput Biol.

[pbio-1000013-b059] Comet I, Savitskaya E, Schuettengruber B, Negre N, Lavrov S (2006). PRE-mediated bypass of two Su(Hw) insulators targets PcG proteins to a downstream promoter. Dev Cell.

[pbio-1000013-b060] Martin D, Brun C, Remy E, Mouren P, Thieffry D (2004). GOToolBox: functional analysis of gene datasets based on Gene Ontology. Genome Biol.

