# Supplementary material for: Functional Anatomy of Polycomb and Trithorax Chromatin Landscapes in Drosophila Embryos
Source: PLoS Biol. 2009 Jan 13;7(1):e1000013. doi: 10.1371/journal.pbio.1000013 (PMC2621266; doi:10.1371/journal.pbio.1000013)
Supplement: Supplementary file 1 [file pbio.1000013.sd001.doc]

**Functional Anatomy of Polycomb and Trithorax Chromatin Landscapes in *Drosophila* Embryos**

**Text S1**

Bernd Schuettengruber^1*^, Mythily Ganapathi^1,4 *^, Benjamin Leblanc^1^, Manuela Portoso^1^, Rami Jaschek^2^, Bas Tolhuis^3^, Maarten van Lohuizen^3^, Amos Tanay^2^, and Giacomo Cavalli^1,5^

^1^ Institut de Génétique Humaine, CNRS, 141, rue de la Cardonille, 34396 Montpellier Cedex 5, France

^2^ Department of Computer Science and Applied Mathematics, The Weizmann Institute of Science, Rehovot, Israel.

^3^ Division of Molecular Genetics, and the Centre for Biomedical Genetics, The Netherlands Cancer Institute, Plesmanlaan 121, 1066 CX Amsterdam, The Netherlands

^4^ present address: Department of Molecular Biology & Biochemistry, Rutgers State University of New Jersey, Nelson Laboratories A123-5, 604 Allison Road, Piscataway, NJ 08854, USA

^*^ these authors contributed equally to the work

Running title: Anatomy of Polycomb and Trithorax Chromatin

Keywords: Polycomb group, Trithorax group chromatin profiling, ChIP on chip, *Drosophila*, PRE.

^5^ Author for correspondence: Giacomo Cavalli

Phone:	+33 499619971

Fax:	+33 499619901

Email:	[Giacomo.Cavalli@igh.cnrs.fr](mailto:Giacomo.Cavalli@igh.cnrs.fr)

**Supporting Results and Discussion**

**Identification of embryonic PcG target genes, gene structure features and Gene Ontology analysis**

By using the criteria of proximity between the PH binding sites and the neighbouring genes (see below: Supporting material and methods), we identified 228 genes that were targeted by PH. 63.8% of these genes overlapped with Schwartz et al [1]. Unique targets in cells or embryos might represent tissue specific PcG targets. Functional classification of the 243 PcG target genes (See methods) revealed that the molecular function GO terms ‘transcription factor’, ‘transcription regulator’ and ‘DNA binding’ activities were significantly enriched while ‘catalytic’, ‘transferase’ and ‘hydrolase’ activities were significantly depleted (Figure S19, Tables S14 to S16). Genes encoding transcription regulators being enriched in PcG target genes has been shown earlier in other genome-wide mapping studies in *Drosophila* cell lines [1,2].

The average length of PcG target genes (~13444bp) was significantly higher as compared to the average gene length in fly genome (~4879bp, two tailed T test p-value 1.61E-12). A comparison of average length of genes localized in H3K27me3 domains (average length ~5236bp, after removal of PcG target genes) to fly genome revealed no significant difference (two tailed T test p-value 0.63). Thus larger average gene length seems to be a unique feature of PcG target genes.

**Over-represented sequence motifs in individual binding profiles**

The over-represented motifs in individual profiles of sequence specific DNA binding factors DSP1, GAF, PHO and PHOL were determined using MEME algorithm [3], see Supporting Information). Only the high confidence ChIP on chip bound regions were taken for this analysis (P-value < 1E-20). The top motif from MEME was tested for its specific enrichment in high confidence ChIP on chip bound regions by comparing its frequency of occurrence to random genomic sequences (Figure 5 and Table S6). The motif presence was also scored in the complete set of ChIP on chip bound sequences (P-value < 1E-04, Table S7). GAF bound regions showed an enriched GA repeat motif of length 15bp (Figure 5). It seems to be a bi-partite motif with the GAGAG (5-mer) prominent on both ends than in the middle. Earlier, both *in vitro* and DamID mapping studies had suggested that GAF binds to clustered GAGAG sites [4,5,6]. The presence of tandem GAGAG repeats as the over-represented motif seems to confirm the above observation. The top motif from DSP1 bound regions contained GA/CT repeats and was similar to GAGA binding motif (Figure 5). We also noticed that the same motif came out to be the most abundant one when we searched the complete set of ChIP on chip bound regions (P-value< 1E-04; SF17). GAF and DSP1 colocalized in a large number of regions in the genome (66% of significantly bound DSP1 regions were also bound by GAF, see Table S4A) and the presence of GAF motifs could have potentially influenced the selection of DSP1 motif. To clarify whether this sequence binds DSP1 or is only a variant of GAGA motif, we analyzed for over-represented motifs in DSP1 bound regions which didn’t show detectable GAF binding (Figure S9). In this new consensus ‘GCGAG’ part of DSP1 motif seems to be conserved, suggesting that this motif might be specific for binding DSP1. A search in JASPAR database to identify any known factor which binds this motif did not reveal significant match. Results from experimental studies are awaited to further confirm the specific binding of DSP1 to this motif.

In both PHO and PHOL regions, a motif similar to the earlier reported PHO binding sequence emerged as the over-represented motif (Figure 5) [7,8]). *In vitro* PHOL is known to bind to the PHO motif [9]. In our data, PHO and PHOL were together bound in large number of regions in the genome (~ 60% of the PHOL sites overlapped with PHO bound regions) and this might influence motif identification. To get clues whether PHO and PHOL bind to the same motif *in vivo*, we separated PHO bound regions wherein PHOL was not detectable (set1; 484 sites) and PHOL bound regions without detectable PHO binding (set2; 30 sites). Set1 showed the enrichment of the previously reported PHO motif but this motif was not enriched in set2 (Figure S10). In fact, the top motif in set2 had a higher E-value, which reflects that this motif could be common in random sequences. A search in JASPAR database to know whether the set2 motif corresponds to any known factor yielded no significant match.

**Over-represented sequence motifs in PH and non-PH regions bound with PcG recruitment factors**

We next determined the over-represented sequence motifs in the complete set of 441 PH peaks. The 300bp (Figure S11A) and 500bp (Figure S11B) sequences centered on the PH peak local maxima of intensity were retrieved and analyzed. The same motifs emerged to be enriched in both the sequence sets. They showed presence of DSP1 and GAF motifs. (CA)-repeats and Poly-T motif also were present in the list. Surprisingly, the PHO motif was not over-represented, even though PHO was bound at all the PH peaks. This could be due to offset of PHO and PH peaks (~50% of cases local maxima of intensity of PHO peaks was shifted/offset to PH peaks). Since the PcG recruitment factors were also present in many genomic regions, which did not have PH binding, we asked whether the underlying sequence determines PH binding to these recruitment factor bound regions. We mined over-represented motifs in 99 PH and 133 non-PH regions (P-value for absence of PH & PC > 0.1), both bound with all recruitment factors (PHO+DSP1+GAF) and compared them (Figure S8). Both regions seemed to be enriched in similar motifs. Comparison between the two sequence sets revealed that GAF/DSP1 like motifs (Figure S8, Amotif 1, Amotif 3 and Bmotif 3) were present in significantly more number of PH bound sites than in non-PH recruitment factor bound regions (Table S5). The density of these motifs was also more in PH sites as compared to non PH regions. The difference in frequency of GAF motifs emerges to be the major distinction between PRE and non-PRE recruiter bound regions. This prompted us to test whether the levels of GAF and the other recruiters were different in PH bound regions than in non-PH bound regions.

**Binding levels of PcG recruitment factors at PH sites**

We examined for differences in the intensities of PcG recruitment factors in PH bound regions as compared to non-PH bound regions (P-value for absence >0.0001). Average binding intensity in PH and non-PH regions were calculated for each factor and were plotted (Figure S12). All recruitment factors except PHOL showed higher binding intensity levels in PH sites as compared to other places in the genome (T test P value for PHO-1.17E-97, DSP1-1.08E-28, GAF-2.01E-10 and PHOL 0.08). Among the factors, PHO showed very high levels of binding at PH regions as compared to non-PH regions. To understand whether frequency of sequence motifs contribute to this higher level enrichment seen at PH sites, we calculated the density of motifs per sequence in both sets (PH and non-PH regions). The motif frequencies were higher in the PH bound regions for PHO, DSP1 and GAF as compared to just non-PH bound regions (Table S8; T test P value for PHO 1.66E-15, GAF 4.63E-09 for DSP1 4.62E-14). This suggests that the density of sequence motifs might be important for efficient recruitment of DNA binding proteins and therefore might be a determinant for PcG recruitment. Other factors such as nucleosome exclusion at PREs might also play a role in enhanced binding of factors at PREs.

These observations suggest that the DNA sequence contains much of the information needed in order to define PREs and TREs. In this case, one should expect to be able to reach the ability to predict PREs. One such attempt was made few years ago, with the publication of a PRE prediction algorithm based on the idea that a PRE may be defined by the clustering of DNA consensus motifs for several of the PcG recruiters, particularly PHO/PHOL, GAF and Zeste. When we compared our binding sites to the predictions, we found that 13.17% of our targets were predicted by Ringrose et al [1,10] (Figure S17). Further, we compared 344 predicted PRE regions of release 4.3 (Rehmsmeier M personal communication, [10,11]) with our 439 ChIP on Chip PH+PC bound regions. 53/344 (15.41%) predicted regions showed PH binding whereas 53/439 (12.07%) of bound PH regions were selected by PREdictor algorithm (Table S9-S11). As reported earlier, we also found that PREdictor was able to predict a low percentage of true positives [1,2]. The algorithm scored better when trying to predict binding sites for PcG recruiters since the number of false positives decreases significantly. However, deeper mining in the sequence features of PcG and trxG targets is needed in order to be able to construct a robust predictive tool to infer their binding pattern at the genome-wide level.

**Supporting Material and Methods**

**Antibodies**

The antibodies for IPs were diluted at 1:100 (PC, PH, H3K27me3, H3K4me3, TRX-C PHO and DSP1), 1:20 (PHOL) and 1:50 (TRX-N) (see also Table S12). Specificity of PHO, PHOL and TRX-C antibodies was confirmed by ChIP (for PHO and PHOL) or Immunostaining (TRX-C) in the corresponding mutant backgrounds (data not shown).

**Preparation of chromatin**

ChIPs were performed on the Oregon-R *w1118* line of *Drosophila melanogaster* (R. Paro - Center of Biosystems, Science & Engineering, Basel, Switzerland). Flies were grown at 25°C on a standard medium for amplification and egg laying was done for 4 hours on plates with standard vinegar medium. Embryos were collected in EWB (Embryo Wash Buffer - 0.03% Triton X100, 0.4% NaCl) 12hr after the beginning of egg laying (4-12hr old embryos). The ChIP method was an adaptation of a previously published protocol [12]. Embryos (approximately 100 mg of embryos per IP) were cross-linked in 5ml buffer A1 (60mM KCl, 15mM NaCl, 15mM HEPES (pH 7.6), 4mM MgCl_2_, 0.5% Triton X-100, 0.5mM DTT and complete EDTA-free protease inhibitor cocktail [ROCHE]), in the presence of 1.8% formaldehyde, by homogenization in a Tenbroeck and then in a Dounce homogenizer with type A pestle (three strokes each), followed by incubation for 15 min at room temperature. Cross-linking was stopped by adding 225mM glycine followed by incubation for 5 min. The homogenate was transferred to a 15-ml tube and centrifuged for 5 min, 4000g at 4°C. The supernatant was discarded and the crude nuclei pellet was washed three times in 3ml A1 buffer and once in 3ml of A2 buffer (140mM NaCl, 15mM HEPES (pH 7.6), 1mM EDTA, 0.5mM EGTA, 1% Triton X-100, 0.5 mM DTT, 0.1% sodium deoxycholate and protease inhibitors) at 4°C. After the washes, nuclei were resuspended in A2 buffer in the presence of 0.1% SDS and 0.5% N-lauroylsarcosine and incubated for 30 min on a rotating wheel at 4°C. Chromatin was sonicated using a Bioruptor (Diagenode) for 10 min (settings 30 sec on, 30 sec off, high power). Sheared chromatin had an average length of 500 to 1000 bp. After sonication and 10 min high speed centrifugation, fragmented chromatin was recovered in the supernatant. Cross-linked chromatin was loaded on CENTRICON YM-100 columns (AMICON cut-off 100kDa) and washed with 3 volumes of A2 buffer in presence of 0.05% SDS (referred as A3 buffer) at 4°C to eliminate uncross-linked proteins and small DNA fragments.

**Chromatin Imunoprecipitation (ChIP) experiments**

Chromatin was pre-cleared by addition of 100ul of Protein A-Sepharose suspension (GE Healthcare, 50% w/v, called PAS) followed by overnight incubation at 4°C. PAS was removed by centrifugation, antibodies were added to the supernatant (a control in the absence of antibody called ‘Mock IP’ was performed at the same time) and samples were incubated for 4 hours at 4°C in a rotating wheel. 50ul of PAS was added and incubation was continued overnight at 4°C. Antibody-protein complexes were collected by centrifugation at 4000rpm for 1 min and the supernatants were discarded. Samples were washed four times in A3 buffer and twice in 1mM EDTA, 10mM Tris (pH 8) (TE) buffer (each wash 5 min at 4°C). Chromatin was eluted from PAS in two steps; first in 100ul of 10mM EDTA, 1% SDS, 50mM Tris (pH 8) at 65°C for 15 min, followed by centrifugation and recovery of the supernatant. PAS material was re-extracted in 150ul of TE, 0.67% SDS. The combined eluate (250ul) was incubated overnight at 65°C to reverse cross-links and treated with Proteinase K for 3h at 50°C. 0.44 LiCl was added to the samples, phenol–chloroform extracted and ethanol precipitated in the presence of 20ug glycogen. DNA was resuspended in 30ul of water. 5ul of the immunoprecipitated DNA was used to validate enrichment of specific DNA fragments by real time PCR (see Figure S13). The remaining 25ul was treated with 10Units of Polynucleotide Kinase (PROMEGA) for 30 min at 37°C. Staggered DNA ends were repaired by incubation with 5 Units of Klenow fragment polymerase (PROMEGA) for 30 min at 37°C in the presence of 2mM of dNTPs. Linkers were ligated to the DNA and Linker-mediated (LM) PCR was performed as previously described [13]. To verify linearity of the LM PCR, amplified DNA was tested by Southern blot analysis using genomic DNA fragments containing known PcG binding sites [12]. 4μg of each amplified sample was sent to Nimblegen Systems Inc. for hybridization on the microarrays.

**ChIP on Chip validation**

ChIP on Chip enrichments were validated by chromatin immunoprecipitation followed by quantitative PCR for specific regions (Figure S13).

**Microarray details**

**Array design**

The 3 x 380K tiling microarrays representing the euchromatic, non-repetitive regions of *Drosophila melanogaster* genome sequence (Flybase release 4.3) from Nimblegen Systems Inc. (GEO accessions: GPL3352, GPL3353 & GPL3354) were used to hybridize the ChIP samples. This set of 3 tiling arrays have in total 909,279 oligonucleotide probes with an average oligonucleotide length of 50bp and an average inter-oligo spacing of 130bp. Additionally, in these arrays, 9 regions have been tiled at higher oligonucleotide density. This data has not been included in our analysis.

The first replicate of all the ChIP samples were hybridized on 3 x 380K tiling microarrays. The second replicate of all the samples except PHO, PHOL and DSP1 were hybridized using the 3 x 380K microarray set; whereas PHO, PHOL and DSP1 samples were hybridized using the 2.1M Nimblegen array (Design ID 6262). This design is inclusive of the 909,279 oligonucleotides (as in the 3 x 380K arrays) in a single array, as well as includes similar number of oligonucleotides for the bottom strand. The data of the bottom strand was not included in our analysis.

**Data analysis**

Whole genome binding profiles of PC, PH, TRX-N, TRX-C, PHO, PHOL, GAGA, DSP1 and of two histone modifications – H3K27me3, H3K4me3 were obtained by combining the data from two biological replicates. Specific (Cy5) and Mock IP (Cy3) signal intensities as quantified and provided by Nimblegen were converted to log2 ratio and normalized using loess normalization [14]. This was done individually for each array. For each profile, normalized log2 ratio of the two replicate samples were then combined by averaging the values at corresponding genomic positions.

**Correlation between biological replicate samples**

The probe-wise correlations between the biological replicate samples are shown in Figure S1. The correlation between the IP samples of two biological replicates and the correlation between two mock samples has been evaluated in Figure S2. A list of all significantly enriched regions (P- value < 0.0001) for all profiles are shown in Table S17.

**Calculating significantly enriched regions**

To calculate whether the levels of enrichment are statistically significant for each array, a normal distribution was calculated, with the assumption that the mode and median absolute deviation of the normalized log2 ratios are average and the standard deviation of the normal distribution, respectively. Assuming that the normal distribution covers the entire background noise (non-significant signals), a p-value was calculated for each oligonucleotide signal. For the two replicate samples of each profile, each pair of probe p values were then combined using a Chi Square law with 4 degrees of freedom. Finally, correction for multiple testing [15] was applied to the combined p-values.

Only oligonucleotides with final p-values (replicates combined) <1E-04 were considered to be significantly enriched for the signal. Enriched regions for PH, GAF, PHO, PHOL, DSP1, TRX-N, TRX-C, and H3K4me3 were calculated by stitching the coordinates of the oligonucleotides which were significantly enriched. This was done after applying a criterion that a minimum of 3 consecutive oligonucleotides should have significant p-values. To define co-localized/overlapping regions, two regions were compared and if they were overlapping even by a single bp they were taken to be co-localised.

**H3K27me3 and PC domains**

PC and H3K27me3 were seen to bind large regions. Intermittently in many of these regions, the signal intensities dropped low (but were distinct than the background) and often such probes didn’t cross the p-value threshold. Hence for these profiles, we applied a criterion that, if two enriched regions (minimum 3 consecutive oligonucleotides with significant p-values) were spaced within <17 non-significant oligonucleotides (~2Kb) then they were stitched into one. Stitching of the gaps reduced the number of regions which were split into two, but still many were left out as they had gaps of >2Kb. Hence, according to visual inspection of the data plots (from the viewer), gaps were stitched when they seemed to be part of a bigger region, even if a gap of >2Kb was separating them apart. These regions have been denoted as ‘domains’ in this manuscript. H3 We observed that larger domains generally contained multiple PH peaks and the intensities of H3K27me3 and PC seemed to be higher in these regions while the intensity in H3K27me3 domains with only one or without PH binding site was generally low.

**H3K27me3 domain features**

ChIP on chip data from S2 cells [1] were downloaded from Array Express (SGR files). In this dataset PSC was reported to bind localised regions alike PH; PC and H3K27me3 were bound to larger regions [1]. The significantly bound regions for PC and H3K27me3 were determined by considering the probes which had intensity ratio of IP/input ≥ 2 ([1]; minimum 3 consecutive probes should have crossed the intensity threshold). If two PC or H3K27me3 enriched regions (minimum 3 consecutive oligonucleotides with significant p-values) were spaced < 2Kb distance then they were stitched into one. For PSC, we took IP/input ratio of ≥ 2.2 and took a gap criterion of < 2Kb [1]. PSC was bound in 263 regions whereas for H3K27me3 was present at 1246 regions. Out of 263, 195 PSC peaks localized in H3K27me3 bound regions.

**PcG target genes**

We first mapped the PH peaks to domains wherein both H3K27me3 and PC were present. Several of these peaks localized to genes which were taken as ‘Genic PcG targets’. For the rest of the PH peaks, which localized to intergenic regions, we took the nearest gene present within the H3K27me3 domain as the target gene. Some of the PH peaks were only present in PC domains and these regions were devoid of significant H3K27me3 binding. The nearest genes to these PH peaks were taken as the target and were denoted as ‘Weak PcG targets’.

**Functional classification**

The genes were functionally classified using GO toolbox ([16]; http://crfb.univ-mrs.fr/GOToolBox/index.php). The ‘molecular function’ ontology, ‘Hypergeometric’ statistical test option and ‘Benjamini and Hochberg’ correction for multiple testing, options were used for the classification. All the genes of *Drosophila melanogaster* genome (inclusive in the GO toolbox web version) were taken as the reference set for functional classification.

**Enriched sequence motifs**

*Identification of over-represented sequence motifs in ChIP on chip bound regions of PcG recruitment proteins (GAF, DSP1, PHO and PHOL)*

Sequence motifs enriched in ChIP on chip bound regions of all the sequence specific binding factors, was determined by the following method. Firstly, the coordinates of the high confidence ChIP on Chip bound regions (P-value <1E-20) were retrieved. In these regions the probe with maximum intensity was determined. The start (bp) position of this probe was taken as the ‘Local maxima of intensity’ (Lmax) of that region. The 300bp sequence, centered on the Lmax of each region was retrieved from the genome (Lmax-150bp to Lmax+150bp; Flybase release 4.3). The sequences were repeat masked using web version of Repeatmasker ([www.repeatmasker.org](http://www.repeatmasker.org/); A.F.A. Smit, R. Hubley & P. Green, unpublished data. Current Version: open-3.1.9 (RMLib: 20071204)). The repeatmasked sequences were taken as input for MEME (Multiple EM for motif Elicitation; [3]; Version MEME - 3.5.4) to search for enriched motifs. It was taken care that the unmasked part of the sequence was >200bp and sequences which had < 200bp of unmasked region (without repeats) were not included for the analysis.

The ‘one occurrence per sequence’ (OOPS) option in MEME was applied for motif identification. For optimizing the motif width parameter we tested 2 options - the default parameter for motif width (Figure 5A) and with motif length specified as 5-10bp (minimum-maximum motif length cut-off, respectively; Figure 5B).

MEME program gives as output a consensus sequence and a motif weight matrix. A motif defined by the MEME program is not just a consensus sequence, but a position-specific probability matrix, which has probabilities associated with each base at each position. MEME motif E-value is the expected number of motifs with equal or higher likelihood, with the same width and number of occurrences, in a similarly sized set of random sequences with same nucleotide composition as the input sequence set.

Further, MAST (Motif alignment and search tool -<http://meme.sdsc.edu/meme/website/meme-download.html>; [17]) which needs MEME output as input was used to count the number of sequences with motifs in MEME input as well as in two sets of control sequences. Fisher’s exact tests were done to test the association of the motif with specific binding profile as compared with control sets (Table S6).

*Control sequences*

Two sets of control sequences were taken for the MEME analysis - 1) the regions wherein none of our profiles (PC, PH, DSP1, GAF, PHO, PHOL, H3K27me3, H3K4me3, TRX) showed binding, were delineated and the sequences were retrieved from the genome. These regions had P-value >0.1 for all the profiles. 10 random sets of sequences similar in length (bp) and number (no. of sequences) to the MEME input were generated from these regions. 2) These control sequences were randomly drawn from the complete genome sequence. They were also similar in length and number to the MEME input sequences. The sequences were masked for repeats before being taken as input for MAST. For each binding profile, 10 sets of random sequences each from control1 and 2 were analysed by MAST for motif distribution.

*Scoring motifs in complete set of ChIP on Chip bound regions*

The motifs present in the complete set of ChIP on Chip bound regions were scored using Patser version 3e [18]. The position specific probability matrix from MEME was used as input for Patser. For the ‘A’ option the (A+T) and (G+C) content of the input sequence set was used. The ‘ln (numerically calculated cutoff p-value)’ given in the Patser output was taken to calculate the threshold P-value for scoring the presence of the motif (all the matches < the given ‘ln numerically calculated cutoff p-value’ were taken).

*Over-represented motifs in DSP1 bound regions wherein significant GAF binding was not detected*

77 genomic regions were retrieved wherein DSP1 was significantly bound (P-value < 1E-04) and GAF was not detected (P-value > 0.1). For all these regions the 300bp sequence centered on Lmax was retrieved, masked for repeats and was taken as input for MEME. The ‘ZOOPS’ (Zero or One Occurrence per Sequence) model and ‘motif width’ as 5-10bp were the input parameters for MEME.

*Over-represented motifs in PHO bound regions wherein significant PHOL binding was not detected and vice-versa*

484 regions were present wherein PHO was significantly bound (P-value < 1E-04) and PHOL was not detected (P-value > 0.1). Only 30 regions were found wherein PHOL was bound (P-value < 1E-04) and PHO was not detected (P-value > 0.1). For all these regions the 300bp sequence centered on Lmax was retrieved, masked for repeats and was taken as input for MEME. The ‘ZOOPS’ (Zero or One Occurrence per Sequence) model and ‘motif width’ as 5-10bp were the input parameters for MEME.

*Motif analysis in PH and non–PH bound regions with PcG recruitment factors*

The PH bound regions wherein all the PcG recruitment factors were bound (PH+PC+DSP1+GAF+PHO; P-value <1E-04) and the non-PH bound regions (neither PH nor PC were detected, P-value >0.1) wherein DSP1, GAF and PHO were significantly bound (P-value <1E-04) were retrieved. The ‘ZOOPS’ model and ‘motif width’ as 5-10bp were taken as input parameters for MEME. All the motifs which came out were counted in both sequence sets using Patser.

*Sequence logos*

Sequence logos for motif consensus were constructed by aligning motifs from MEME input sequences using Weblogo server (http://weblogo.berkeley.edu/logo.cgi; m[19]).

Motif matrices were searched against JASPAR database (<http://jaspar.genereg.net/>) to identify the corresponding binding factors [20].

**Comparison with Ringrose et al (2003) and Rehmsmeier et al (2006) predictions**

To evaluate the overlap of predicted regions with actual ChIp on chip binding, we compared the 344 predicted PREs ([10,11]; release 4.3 predictions; Rehmsmeier M personal communication) along with our 439 experimentally determined regions which showed simultaneous binding of PH and PC.

As the predictions were based on occurrence of pair-wise combinations of sequence motifs of DNA binding proteins, involved in PcG recruitment, we compared the predicted PRE regions to the regions bound by PHO, PHOL, DSP1 and GAF individually as well as in combination. We also compared the PREdictor scores of positively predicted regions (predicted as well as bound) versus negatively predicted regions (predicted but not bound) to assess whether higher scoring PREdictor regions are more frequently bound by PcG proteins in chip on chip data.

**Average intensity of PcG recruitment factors in PH and non-PH bound regions.**

To test whether a protein, for instance GAF, shows a stronger enrichment in PH sites as compared to non-PH sites, we consider the set of sites strongly enriched for GAF in the whole genome (P-value < 1E-04). We then compare the average signal intensity per site in two distributions, one for GAF in the subset of sites showing a strong PH enrichment denoted{GAF AND PH} and for GAF in the rest of GAF sites, denoted {GAF AND NOT PH}. Sites were always selected according to probe P-values. For example, {GAF AND PH} sites are genomic regions with at least 3 consecutive probes, each one having P-values for GAF and PH both below the threshold chosen in our analysis (1E-04). {GAF AND NOT PH} sites are all regions showing at least 3 consecutive probes with a combined P-value >1E-04 for PH and < 1E-04 for GAF. Average signal intensity per selected site was calculated taking the sum of all the log2 ratios divided by the number of probes for each considered site.

**PHO-PHOL binding at PH sites**

The PHO-PHOL ratio for the whole genome data was calculated as follows. The global PHO and PHOL distributions of log2 ratios of intensities were first individually centered and reduced so that both the distributions had mean = 0 and variance = 1. The log2 ratio of each PHO probe was then subtracted by log2 ratio of the corresponding PHOL probe. This per probe value which corresponds to PHO-PHOL ratio was used to generate a genomic profile which can be visualized in the viewer.

To analyse PHO-PHOL ratio in PH and non-PH bound regions we did the following. The genomic regions wherein PHO or PHOL were significantly bound (union of PHO or PHOL sites) were taken. These regions were divided into 2 sets, namely, ‘PHO OR PHOL AND PH’ (showing at least 3 consecutive probes with P-value <1E-04 for PHO OR PHOL and PH) and ‘PHO OR PHOL AND NOT PH’ (3 consecutive probes with P-value <1E -04 for PHO OR PHOL and P-value >1E-04 for PH). Individual PHO-PHOL log2 ratios of all the probes in a region were calculated by the above mentioned method and were averaged to obtain a PHO-PHOL ratio for that region. We also calculated average PHO log2 ratios in ‘PHO OR PHOL AND PH’ and ‘PHO OR PHOL AND NOT PH’ sets. The same was also done for PHOL. Since both PHO and PHOL have been shown to bind the same DNA motif in vitro hence we took the whole set of PHO or PHOL bound regions were taken for the analysis.

**Zeste binding sites**

The Zeste genome-wide ChIP on chip data of fly embryos was downloaded from <http://bdtnp.lbl.gov/Fly-Net/SearchChipper?first=23> (file named - z_2_051504-sym-1_table). The ChIP on chip data for Zeste was reported by Moses et al [21]. The authors had recently re-analysed their data according to updated methods which have been described in (Li et al. 2008). This re-analysed data was downloaded from the above mentioned website and used for our comparisons.

**Hierarchical spatial clustering**

We perform hierarchical spatial clustering as described below (a non hierarchical version was used in [22]). The spatial clusters model is an HMM on **k** states, where each state represents a cluster and is defined by a multivariate distribution on the analyzed experimental tracks. **k** is determined heuristically. In the present study we modeled the emissions of each track from each state using a normal distribution, assuming that given the state, the tracks are mutually independent. The parameters defining the emissions from the model were therefore including the means μ_i_^j^ and std deviations σ_i_^j^ for each state i and each track j. The spatial structure of the model was defined by the HMM transition probabilities between states (Figure S18). We restrict the topology of transition between states to prevent over fitting. In the model used here, the topology forced a hierarchical model in which groups of HMM states were associated together to form super clusters.

We learn a spatial cluster model automatically from the data using the EM algorithm. We initialize the EM using random selection of states parameters from an initial pool of *seeds*. To define the seeds, we use a Z-score threshold (1.5) to transform the values of each probe to -1,0 or 1. Each seed is then defined by a combination of discrete values. We then select a pool of fifty seeds that are the most frequent in the data. This initialization allows the EM to start from defined combinations of marks and factors occupancies that occur frequently in the data. The EM algorithm then optimize the parameters until reaching a local maximum. We test 100 initial seeds and select the solution with the maximum likelihood.

To learn hierarchical models, we use a top down approach. We first learn a 3-states model on the entire data, generating "super-clusters". We perform inference (forward-backward decoding) to determine the posterior state probabilities for each probe. We use these posteriors to partition the genome into three, according to the posterior probabilities of the association between super-clusters and probes. We then retrain a larger HMM model in which each super cluster is substituted by several states (the number is determined by the algorithm randomly). While learning the larger set of parameters, we force the original partition of the genome (so only states that are part of the original super cluster can emit the probe data with non zero probability). To generate the result reported here, we used a 10-states HMM, and derive a maximum likelihood model that contained 4 states for the H3K27me3 and H3K4me3 super cluster and 2 states for the background super cluster.

**Finding discriminating motifs**

To discover motifs that discriminate among spatial clusters, we extended an algorithm for identifying discriminating PWMs [23] by allowing multiple sets to be analyzed together. The input for the algorithm was generated by applying the spatial cluster model to classify genomic loci according to their associated state (the HMM state with the highest posterior probability for that locus). We identified contiguous genomic intervals in which all probes were significantly associated with a specific cluster (posterior probability > 0.5). We then extract 500bp around the center of this interval and tag it with the state. The result was a collection of thousands of sequences tagged by one of 10 states.

The motif algorithm included two phases: scanning and refinement. In the scanning phase we compute a statistical score for all DNA k-mers (k equals 5-8). Instead of using a hypergeometirc p-value as described before, we have computed a chi-square p-value by testing the distribution of k-mer frequencies in each of the clusters' sequence sets. Whenever a significant p-value was detected, we divided the clusters set into high frequency sets (over 1.3 times the expected number of k-mer appearances), low frequency sets (less than 0.8 times the expected number of k-mer appearances) and neutral sets. We then apply the refinement phase as described before (using an EM-like algorithm that tries to maximize a hypergeometric p-value), but using the union of high frequency set as positives and the union of low frequency sets as negatives, disregarding the neutral sets. To eliminate repeated detection of similar motifs from overlapping k-mer seeds, we used masking of high likelihood sequence matching of inferred PWM with significant scores, such that the next seed refinement could not use the same loci for training.

**Computing predicted binding energies**

Given a PWM, we compute the predicted binding energy [24] as log(Σ_j_(Πw_i_[s[j+i]])), where w_i_[c] is the PWM probability for occurrence of character c at position i. To generate figure 6, we used predicted energies in 100bp sequences around each probe centers. To polarize clusters' sequences (selecting a strand) we used the UCSC genome annotation to detect the strand of the nearest TSS, reversing the profile whenever the TSS was associated with the minus strand.

**Supporting references**

1. Schwartz YB, Kahn TG, Nix DA, Li XY, Bourgon R, et al. (2006) Genome-wide analysis of Polycomb targets in Drosophila melanogaster. Nat Genet 38: 700-705. Epub 2006 May 2028.

2. Tolhuis B, de Wit E, Muijrers I, Teunissen H, Talhout W, et al. (2006) Genome-wide profiling of PRC1 and PRC2 Polycomb chromatin binding in Drosophila melanogaster. Nat Genet 38: 694-699. Epub 2006 Apr 2020.

3. Bailey TL, Elkan C (1994) Fitting a mixture model by expectation maximization to discover motifs in biopolymers. Proc Int Conf Intell Syst Mol Biol 2: 28-36.

4. Omichinski JG, Pedone PV, Felsenfeld G, Gronenborn AM, Clore GM (1997) The solution structure of a specific GAGA factor-DNA complex reveals a modular binding mode. Nat Struct Biol 4: 122-132.

5. Katsani KR, Hajibagheri MA, Verrijzer CP (1999) Co-operative DNA binding by GAGA transcription factor requires the conserved BTB/POZ domain and reorganizes promoter topology. Embo J 18: 698-708.

6. van Steensel B, Delrow J, Bussemaker HJ (2003) Genomewide analysis of Drosophila GAGA factor target genes reveals context-dependent DNA binding. Proc Natl Acad Sci U S A 100: 2580-2585. Epub 2003 Feb 2524.

7. Mihaly J, Mishra RK, Karch F (1998) A conserved sequence motif in Polycomb-response elements. Mol Cell 1: 1065-1066.

8. Fritsch C, Brown JL, Kassis JA, Muller J (1999) The DNA-binding polycomb group protein pleiohomeotic mediates silencing of a Drosophila homeotic gene. Development 126: 3905-3913.

9. Brown JL, Fritsch C, Mueller J, Kassis JA (2003) The Drosophila pho-like gene encodes a YY1-related DNA binding protein that is redundant with pleiohomeotic in homeotic gene silencing. Development 130: 285-294.

10. Ringrose L, Rehmsmeier M, Dura JM, Paro R (2003) Genome-wide prediction of Polycomb/Trithorax response elements in Drosophila melanogaster. Dev Cell 5: 759-771.

11. Fiedler T, Rehmsmeier M (2006) jPREdictor: a versatile tool for the prediction of cis-regulatory elements. Nucleic Acids Res 34: W546-550.

12. Negre N, Lavrov S, Hennetin J, Bellis M, Cavalli G (2006) Mapping the distribution of chromatin proteins by ChIP on chip. Methods Enzymol 410: 316-341.

13. Cavalli G, Paro R (1999) Epigenetic inheritance of active chromatin after removal of the main transactivator. Science 286: 955-958.

14. Cleveland WS, Kleiner B, Warner JL (1976) Robust statistical methods and photochemical air pollution data. J Air Pollut Control Assoc 26: 36-38.

15. Benjamini Y, Drai D, Elmer G, Kafkafi N, Golani I (2001) Controlling the false discovery rate in behavior genetics research. Behav Brain Res 125: 279-284.

16. Martin D, Brun C, Remy E, Mouren P, Thieffry D, et al. (2004) GOToolBox: functional analysis of gene datasets based on Gene Ontology. Genome Biol 5: R101.

17. Bailey TL, Gribskov M (1998) Methods and statistics for combining motif match scores. J Comput Biol 5: 211-221.

18. Hertz GZ, Stormo GD (1999) Identifying DNA and protein patterns with statistically significant alignments of multiple sequences. Bioinformatics 15: 563-577.

19. Crooks GE, Hon G, Chandonia JM, Brenner SE (2004) WebLogo: a sequence logo generator. Genome Res 14: 1188-1190.

20. Sandelin A, Alkema W, Engstrom P, Wasserman WW, Lenhard B (2004) JASPAR: an open-access database for eukaryotic transcription factor binding profiles. Nucleic Acids Res 32: D91-94.

21. Moses AM, Pollard DA, Nix DA, Iyer VN, Li XY, et al. (2006) Large-scale turnover of functional transcription factor binding sites in Drosophila. PLoS Comput Biol 2: e130.

22. Gal Yam E, Egger G, Zhang X, Lin J, Liang G, et al. (2008) Frequent switching of Polycomb repressive marks and DNA hypermethylation in the PC3 prostate cancer cell line. PNAS In press.

23. Tanay A, Regev A, Shamir R (2005) Conservation and evolvability in regulatory networks: the evolution of ribosomal regulation in yeast. Proc Natl Acad Sci U S A 102: 7203-7208.

24. Tanay A (2006) Extensive low-affinity transcriptional interactions in the yeast genome. Genome Res 16: 962-972.
